# Rare Variants in HTRA1, SGTB, and RBM12 Confer Risk of Atherosclerotic Cardiovascular Disease Independent of Traditional Cardiovascular Risk Factors

**DOI:** 10.1161/CIRCGEN.125.005233

**Published:** 2025-11-05

**Authors:** Sam M. Lockhart, Anuradhika Puri, Yajie Zhao, Vladimir Saudek, Eugene J. Gardner, Katherine A. Kentistou, Brian Y.H. Lam, Felix R. Day, Stephen O’Rahilly, John R.B. Perry, Ken K. Ong, Meredith E. Jackrel

**Affiliations:** 1 Wellcome-Wolfson Institute for Experimental Medicine, Queen’s Univ Belfast, Belfast; 2 Metabolic Research Laboratories and NIHR Cambridge Biomedical Research Centre, Institute of Metabolic Science; 3 Dept of Chemistry, Washington Univ, St. Louis, MO; 4 MRC Epidemiology Unit, Institute of Metabolic Science; 5 Dept of Paediatrics, Univ of Cambridge, Cambridge, United Kingdom

**Keywords:** Atherosclerotic cardiovascular disease, HTRA1, CARASIL, Exome wide association study

## Abstract

**Background:**

Atherosclerosis is a pathophysiological process common to a range of cardiovascular diseases. We reasoned that considering clinical presentations of atherosclerosis across the coronary, peripheral and cerebrovasculature as a single entity would enhance statistical power to identify rare genetic variation driving pathological processes across multiple vascular beds.

**Methods:**

We performed an Exome-wide association study of atherosclerotic cardiovascular disease in 434,438 UK Biobank participants of European ancestry.

**Results:**

We identified rare, predicted damaging variants in HTRA1, SGTB and RBM12 to be associated with risk of ASCVD, independent of known risk factors. Both SGTB and HTRA1 were downregulated in the aorta of patients with coronary artery disease compared to controls. LOF variants in the RNA-binding protein RBM12 increased the risk of coronary, cerebrovascular and peripheral vascular disease to a similar extent. SGTB increased risk of ASCVD in the coronary and peripheral circulations, but not the cerebrovasculature. While loss of function variants in HTRA1 are known to cause monogenic stroke syndromes, we found that damaging missense variants in HTRA1 are associated with increased risk of disease in both the cerebrovascular and coronary circulation. Surprisingly, the increased risk of coronary artery disease was driven predominantly by a single missense variant (p.R227W, MAF=0.009). In vitro, the R227W mutant HTRA1 efficiently proteolyzed the disordered substrate casein but not aggregated alpha-synuclein. In contrast, a stroke risk raising variant (D320N) could not efficiently process any of the tested substrates.

**Conclusions:**

We identified novel genetic variants predisposing to ASCVD that act independently of established cardiovascular risk factors. The observed phenotypic and functional heterogeneity between HTRA1 variants suggest distinct biochemical mechanisms drive HTRA1-related vascular disease in the brain and heart.

## Nonstandard Abbreviations and Acronyms

ASCVD: Atherosclerotic cardiovascular disease
CADASIL: Cerebral autosomal dominant arteriopathy with subcortical infarcts and leukoencephalopathy
CARASIL: Cerebral autosomal recessive arteriopathy with subcortical infarcts and leukoencephalopathy
ExWAS: Exome-wide association study
FDR: False discovery rate
LOF: Loss of function
LOFTEE: Loss-of-Function Transcript Effect Estimator
LTBP1: Latent transforming growth factor beta binding protein 1
MAC: Minor allele count
MAF: Minor allele frequency
MI: Myocardial infarction
PC: Principal component
PTV: Protein-truncating variant
REVEL: Rare Exome Variant Ensemble Learner
SAIGE-GENE+: Scalable and Accurate Implementation of Generalized mixed model–Gene-based association test
STARNet: Stockholm-Tartu Atherosclerosis Reverse Network Engineering Task
ThT: Thioflavin T (amyloid-binding dye)
UKBB: UK Biobank

## Introduction

Atherosclerosis causes ischaemic vascular disease in the brain, heart and peripheral vasculature. The development of efficacious prevention strategies targeting cardiovascular risk factors such as hypertension, LDL-cholesterol and other ApoB containing lipoproteins have revolutionized the care of these conditions. However, stroke and ischaemic heart disease remain the leading causes of morbidity and mortality in adults worldwide^1^ and the prevalence and morbidity attributable to peripheral vascular disease is increasing^2–4^. Recent estimates suggest that >40% of cardiovascular disease occurs independently of traditional cardiovascular risk factors ^5^. This data, combined with increasing recognition of ischaemic cardiovascular events occurring in the absence of standard modifiable risk factors^6^, provide renewed impetus to discover novel drivers of atherosclerotic cardiovascular disease^7,8^.

Human genetics is unique in its capacity to identify novel causal drivers of disease in an unbiased fashion and is increasingly used to prioritise therapeutic targets^9^ for drug development. Common variant association studies support biological processes acting directly in the vascular wall as modifiers of cardiovascular risk^10–12^. However, uncertainty around annotation of causal variants and genes means translating these associations to a mechanistic understanding of disease is challenging ^13^. In contrast, assessment of rare coding variation using exome-wide sequencing data can confidently annotate a causal gene^14–17^. This approach has provided insights into a range of cardiometabolic diseases including coronary artery disease, where exome-sequencing based studies have identified low-frequency variants associated with coronary artery disease risk (10%<MAF<1%)^18,19^. However, systematic examination for rare coding variation associated with atherosclerotic cardiovascular disease has met with limited success, potentially due to lack of statistical power^20^.

Traditionally, genetic determinants of atherosclerotic cardiovascular diseases have been studied separately. However, they share a fundamental pathological process and are correlated genetically and observationally. We reasoned that amalgamation of atherosclerotic cardiovascular disease across multiple vascular beds would enhance statistical power to identify genes with a causal role in vascular disease.

To this end, we performed an exome wide association study of atherosclerotic cardiovascular disease (ASCVD) in UK Biobank (UKBB), identifying 3 novel ASCVD risk genes acting independently of traditional cardiovascular risk factors. For one such gene, HTRA1, we find evidence of functional and phenotypic heterogeneity with distinct missense variants raising coronary and cerebrovascular risk. Together our findings provide novel insight into the pathophysiology of atherosclerotic cardiovascular disease and vascular multimorbidity.

## Methods

### Data availability

Rare variant association testing described in this paper was conducted using the MRC-EPID WES pipelines (https://github.com/mrcepid-rap/). All data used in the genetic association analyses are available from the UKBB upon application (https://www.ukbiobank.ac.uk).

### Ethical approval

The UKBB data have approval from the North West Multi-centre Research Ethics Committee as a research tissue bank. All participants provided written informed consent. This research has been conducted using the UK Biobank Resource under Application Number 9905. All analyses reported here were conducted in accordance with relevant ethical guidelines.

Further information regarding methods are provided in the supplemental material.

## Results

### An ExWAS of atherosclerotic cardiovascular disease implicate HTRA1, SGTB and RBM12 in atherosclerosis across multiple vascular beds

To identify novel genetic drivers of ASCVD, we conducted an exome-wide association study for ASCVD in 434,438 UK Biobank participants of European ancestry. ASCVD cases were defined as any participant with evidence of coronary, cerebrovascular, or peripheral vascular disease (N=61,598, case prevalence: 14.2%; Table S1, Methods). Gene-based collapsing tests were performed by aggregating rare (MAF<0.001) variants across 19,457 protein coding genes as described previously^16,17^. Burden testing was conducted using 3 variant masks per gene: Predicted loss of function protein truncating variants (PTVs) as defined by LOFTEE^21^ and predicted damaging missense variants with REVEL scores >0.5 or >0.7^22^. This resulted in a total of 37,686 individual gene x mask combinations and a stringent, Bonferroni-corrected multiple testing threshold of P<1.33 x 10^-6^. Our results appeared statistically well calibrated as evidenced by the absence of any synonymous variant mask associations with ASCVD (included as a negative control) and low exome-wide test-statistic inflation (*λ*_GC_=1.03).

**Figure 1:**
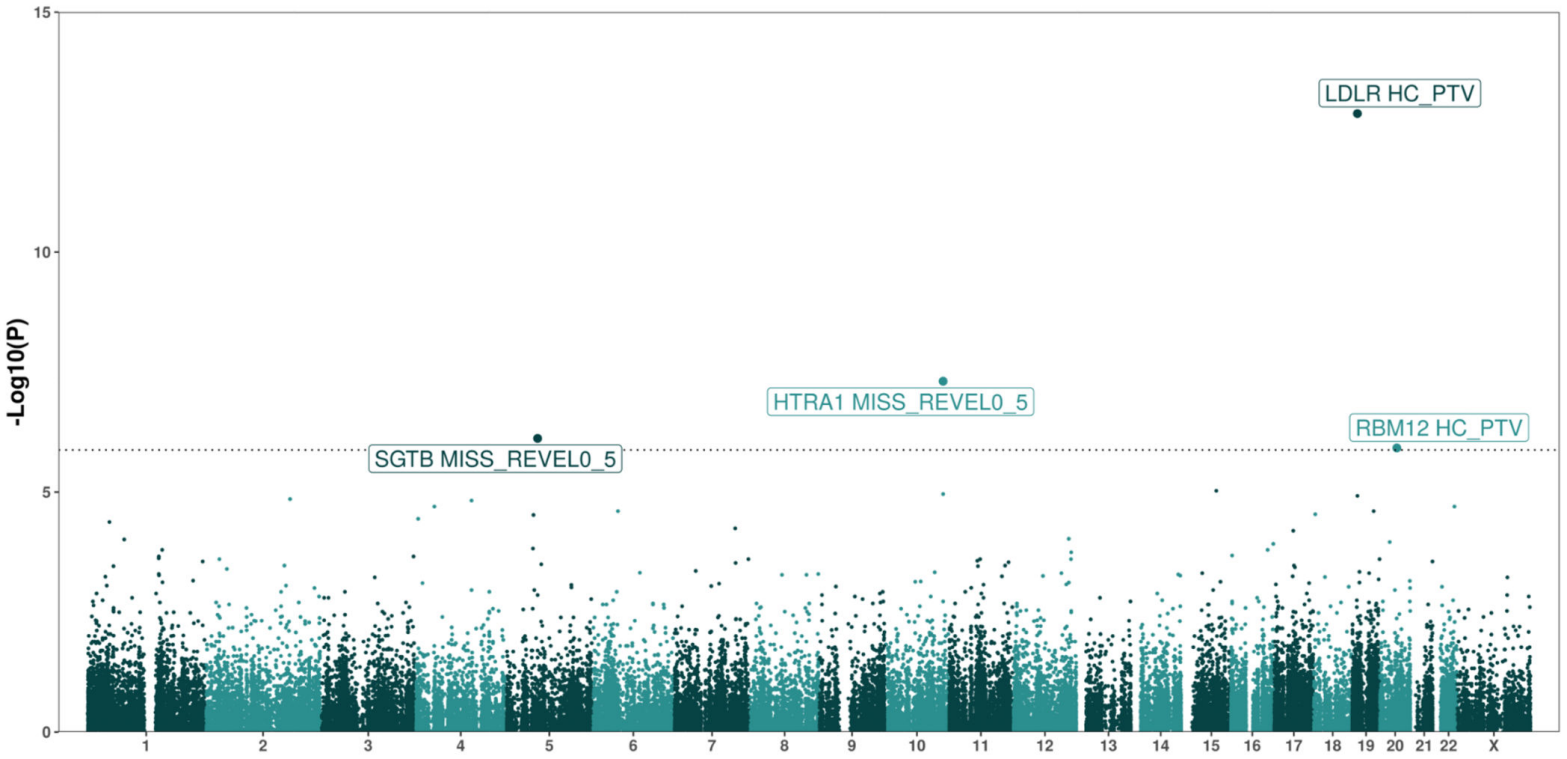
An exome wide association study of atherosclerotic cardiovascular disease in UKBB participants of European ancestry. Manhattan plot summarising an exome-wide association study of atherosclerotic cardiovascular disease in UKBB. Each dot represents an individual gene x mask combination. The dotted line represents a Bonferroni-corrected multiple testing threshold of 1.33 x 10-6. The labels represent gene names where any single gene x mask combination is significantly associated with atherosclerotic cardiovascular disease. Test statistics were derived from a linear-mixed model of European participants in UKBB (implemented in BOLT) with adjustment for age, age-squared, sex, the first 10 genetic principle components and whole exome-sequencing batch.

We identified 4 unique genes associated with ASCVD; LDLR, HTRA1, SGTB and RBM12 (Figure 1, Table S2). Associations with ASCVD were not sensitive to variations in basis of phenotype definitions (e.g. inclusion/exclusion of self-reported cases etc) and leave one out analyses did not suggest any association was driven solely by a single variant (Table S3-S4). We observed similar associations using an orthogonal statistical modelling framework (linear mixed models implemented in SAIGE-GENE+), though P-values were somewhat attenuated compared to those obtained in discovery with BOLT-LMM (Table S14).

**Figure 2:**
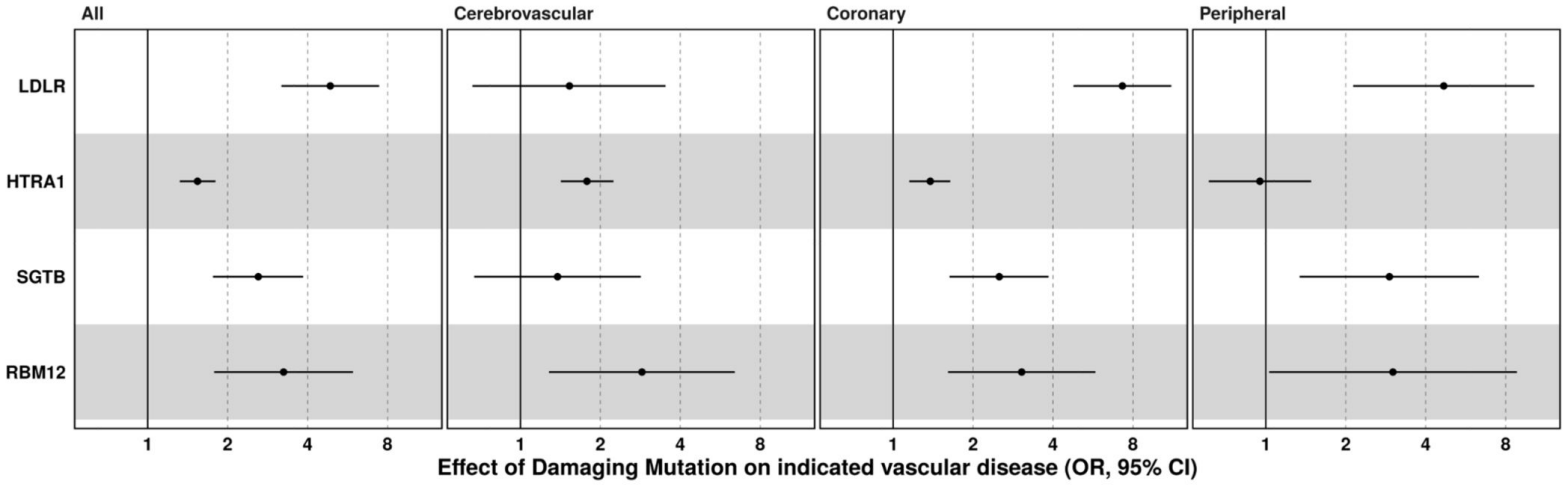
Atherosclerotic cardiovascular disease risk genes increase disease risk via actions in multiple vascular beds. To provide insight into the tissue of action of the candidate genes we partitioned our atherosclerotic cardiovascular disease phenotype into vascular beds and assessed the effect of the most strongly associated mask for each of the three exome-wide significant atherosclerotic cardiovascular risk genes. The test statistics plotted are from a generalised linear model after adjustment for the first 10 genetic principal components, age, age-squared and sex. P-values from collapsing burden tests implemented in BOLT-LMM are available in Table S5. OR: Odds ratio CI: Confidence intervals

LDLR encodes the low density lipoprotein receptor. Monoallelic or bialleleic loss of function variants in LDLR cause familial hypercholesterolaemia and premature onset atherosclerotic cardiovascular disease. Carriers of PTVs in LDLR exhibited an almost 5-fold increase in odds of ASCVD (OR:4.87 95%CI: 3.19,7.44, P=1.30x10^-13^, carriers=75, case prevalence=37%) that was driven predominantly by disease in the peripheral and coronary circulation (Figure 2, Table S5), consistent with existing data on monogenic familial hypercholesterolaemia^23–25^.

HTRA1 is a homotrimeric serine protease that is expressed in endothelial and smooth muscle cells of the vasculature^26–28^. Bi-allelic loss of function variants cause arteriopathy in the cerebral circulation which presents clinically with premature stroke and early onset vascular dementia ^28^. Rare, predicted damaging missense variants (REVEL>0.5) were associated with ~50% increase in the odds of ASCVD (OR:1.54, 95% CI:1.32, 1.80, P=4.9x10^-8^). It is well established that heterozygous carriage of loss of function variants in HTRA1 increases risk of ischaemic stroke and imaging biomarkers of cerebrovascular disease^29–31^. However, this association was driven by both an increased risk of disease in both the coronary (OR:1.38, 95% CI:1.15,1.64, P=5.4x10^-4^) and cerebrovasculature (OR:1.78, 95% CI:1.42,2.24, P= 5.50x10^-7^) (Figure 2, Table S5).

SGTB (Small Glutamine Rich Tetratricopeptide Repeat Co-Chaperone Beta) is a poorly characterised protein that is broadly expressed but exhibits particular enrichment in the brain. Its homologue, SGTA, has a role in regulating localisation and disposal of misfolded proteins^32–34^, but SGTB has not been well studied in any biological context. It is likely that, like SGTA, the N-terminal domain of SGTB facilitates formation of homodimers. It then functions in a chaperone complex where its C-Terminal domain recognises and facilitates interaction with client proteins, modulating their localisation and degradation. Missense variants (REVEL>0.5) in SGTB appeared to raise risk of ASCVD primarily via effects in the coronary and peripheral circulation (Figure 2, OR(Coronary):2.51, 95% CI:1.63, 3.85, P=1.5x10^-5^, OR(Peripheral): 2.92, 95% CI: 1.34, 6.34, P=4.8x10^-3^). Interestingly, the effects of SGTB appeared to be driven by two distinct missense variants (p.SGTB A138V and A228V, OR(ASCVD):2.84, 95% CI:1.83-4.39, P=2.9x10^-6^), one in the highly conserved Tetratricopeptide repeat domain and one in the C-terminal domain which recognises client proteins. While the alanine to valine substitution is relatively mild, both of these residues are extremely well conserved (Figure S1), predicted to be damaging by multiple orthogonal bioinformatic predictors and occur in structurally very important regions buried inside the tightly packed complex. This supports the deleteriousness of these variants and the assertion that damaging variants in SGTB increase ASCVD risk.

Finally, RBM12 is a poorly characterised RNA-binding protein likely implicated in regulation of pre-mRNA splicing^35^. Protein truncating variants in RBM12 appeared to increase risk of vascular disease across all three sub-phenotypes studied with ~3-fold increase in odds of coronary, cerebrovascular and peripheral vascular disease (Figure 2, Table S5).

### Damaging variants in HTRA1, SGTB and RBM12 are not associated with changes in traditional cardiovascular risk factors

**Figure 3:**
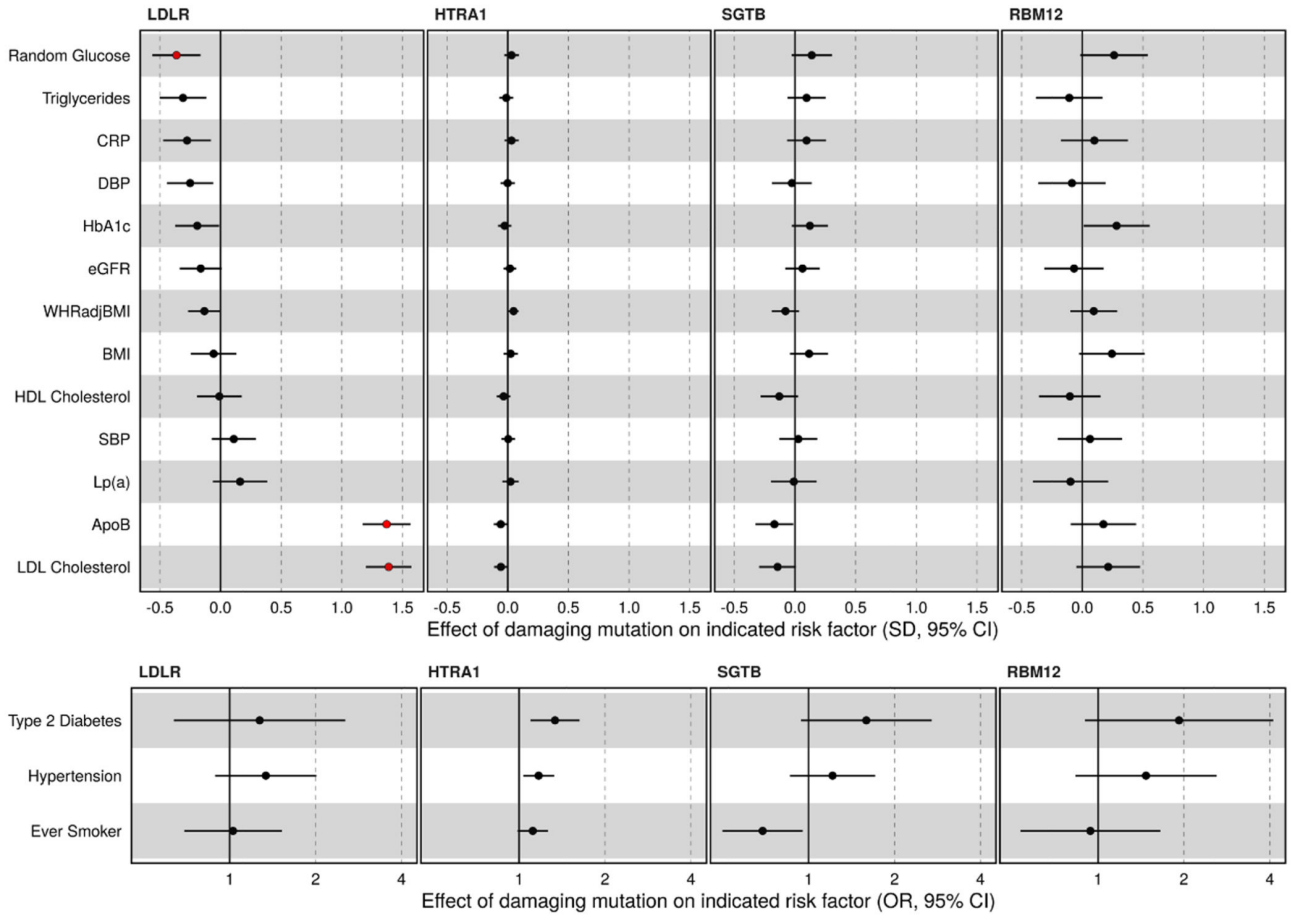
Effects of pLOF variants in atherosclerotic cardiovascular disease risk genes on selected cardiovascular risk factors. The effects of the atherosclerotic risk genes on continuous (Top panel) and dichotomised cardiovascular risk factors (bottom panel) are shown. For each gene the variant mask most significantly associated with atherosclerotic cardiovascular disease is plotted. The points coloured red are significantly associated with the indicated risk factor after adjustment for multiple testing. SD = standard deviation, CI=confidence interval, OR=odds ratio.

Atherosclerosis has a number of well-established causal risk factors which could be driving the effect of identified risk genes. To determine if this was the case we assessed the effect of ASCVD risk genes on a panel of cardiovascular risk factors. As expected, PTVs in LDLR had a large effect on LDL cholesterol and Apolipoprotein-B (Table S7, Figure 3). Consistent with known effects of chronic elevations in LDL cholesterol on glycaemia^36,37^, random glucose was significantly reduced in carriers of LDLR PTVs, though there was no association with type 2 diabetes risk or HbA1c, after adjustment for multiple testing (Table S7-S8, Figure 3). In contrast, there was no significant association with predicted damaging variants in HTRA1, RBM12 or SGTB with any of the tested cardiovascular risk factors, after adjustment for multiple testing. Consistent with effects of damaging variants in HTRA1, SGTB and RBM12 acting independently of traditional cardiovascular risk factors, these associations persisted after adjusting for LDL cholesterol, smoking, Type 2 Diabetes, BMI and systolic blood pressure (Table S9).

### HTRA1 and SGTB are robustly expressed in vascular tissue and are downregulated in patients with coronary artery disease

Given the lack of a convincing association between the novel ASCVD risk genes and established cardiovascular risk factors, we reasoned that these genes could be mediating their effects via direct actions in the vasculature. To assess the biological plausibility of this hypothesis we examined the expression distribution of these genes in the STARNET database which contains transcriptomic data from patients undergoing coronary artery bypass grafting and controls^38^. Consistent with the known biology of HTRA1, it was robustly expressed in the Aorta and mammary artery (Aorta: Median expression (Q1,Q3)=385 (308,475) cpm, Internal mammary artery: Median expression (Q1, Q3)=241 (206,280) cpm). Interestingly, HTRA1 expression was significantly downregulated in aorta from patients with atherosclerotic coronary artery disease versus controls (FDR corrected P-value=3.4x10^-4^). In contrast to the well-described direct actions of HTRA1 in the cerebral vasculature, the biological significance of RBM12 and SGTB in the vascular wall is unknown. Both RBM12 (Aorta: Median expression (Q1,Q3)=123 (110,135) cpm, Internal mammary artery: Median expression (Q1, Q3)=126 (113,139) cpm) and SGTB (Aorta: Median expression (Q1,Q3)=70 (64,78) cpm, Internal mammary artery: Median expression (Q1, Q3)=74 (66,83) cpm) were robustly expressed in aorta and internal mammary artery. While RBM12 was not differentially regulated in aorta from patients with cardiovascular disease, SGTB was significantly downregulated (FDR corrected P-value=2.1x10^-5^). This data is consistent with reduced expression of HTRA1 and SGTB contributing to atherosclerosis in sporadic atherosclerotic cardiovascular disease.

### Predisposition to coronary artery disease is not a general feature of monogenic cerebral small vessel disease

The association of coronary artery disease with predicted damaging variants in HTRA1 (Figure 2) led us to consider if disease of the coronary circulation might be an unrecognised feature of other monogenic cerebral small vessel disease syndromes. CADASIL is an autosomal dominant cerebral small vessel disease syndrome caused by missense variants in NOTCH3 and it shares some of the clinical and pathophysiological features of HTRA1-related cerebrovascular disease^39^, including early-onset stroke and vascular dementia. We examined the relationship between pathogenic (e.g. CADASIL-causing) variants in NOTCH3^29^ and coronary artery disease. As expected, pathogenic variants were associated with an increased risk of cerebrovascular disease (OR=2.14, 95% CI: 1.68, 2.74, P=4.43x10^-10^) (Table S10). However, there was no increased risk of coronary disease (OR=0.79, 95% CI=0.60, 1.03, P=0.08). Thus, the effect of HTRA1 on the coronary vascular health is not a general feature of cerebral small vessel disease and may be related to specific effects of HTRA1 in the coronary vasculature.

### Distinct variants drive HTRA1-related vascular disease in the cerebral and coronary circulation

**Figure 4:**
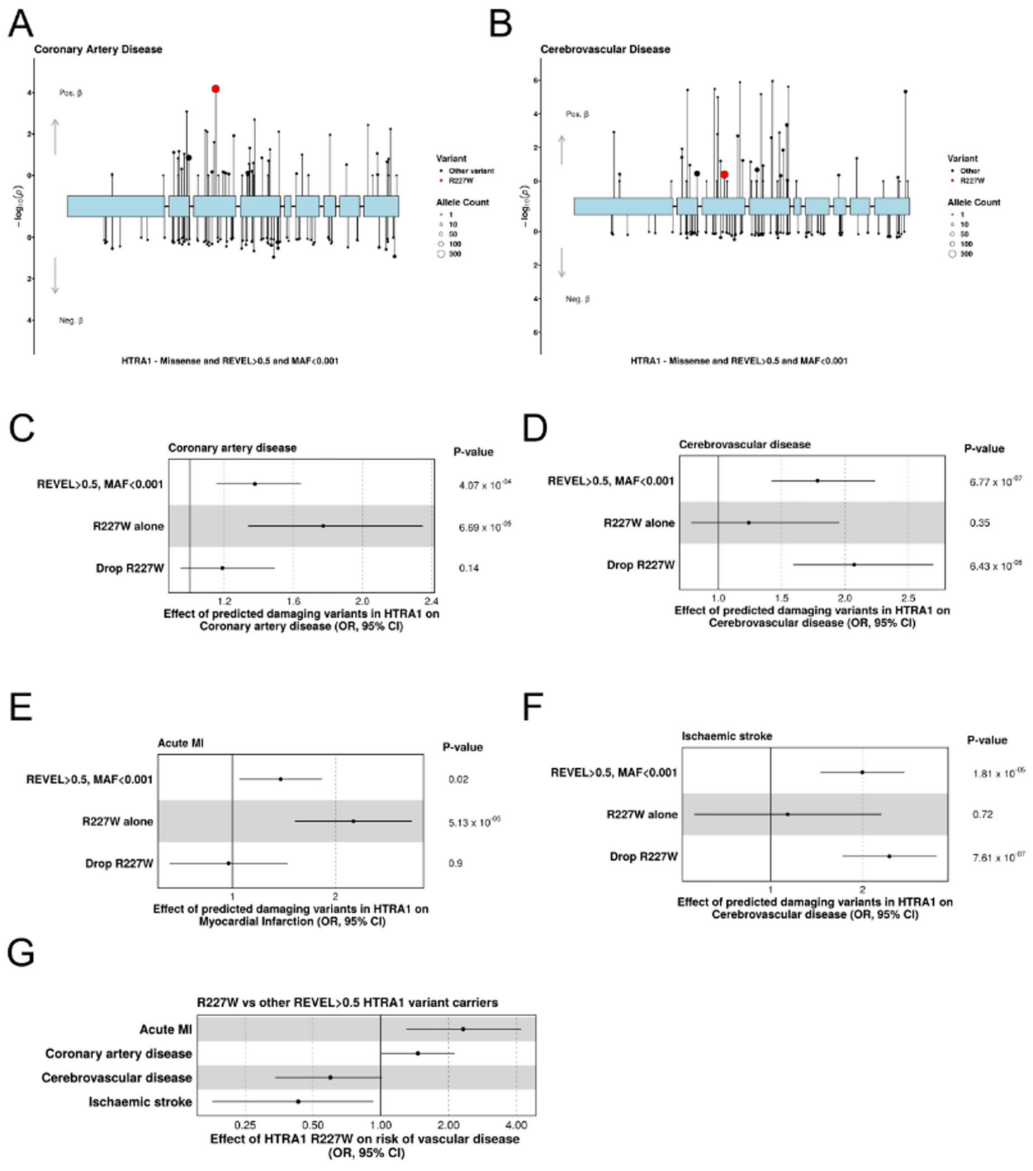
p.R227W increases coronary artery disease risk but not risk of cerebrovascular disease. A+B represent lolliplots of variants in HTRA1 and their effect on coronary artery disease (A) and cerebrovascular disease risk (B). The size of each dot is proportional to number of carriers. The red dot represents p.R227W which has differential effects on cerebrovascular and coronary artery disease. Test statistics plotted were derived from a linear mixed model implemented in BOLT. C-F: Effect of mask composition on coronary artery disease, cerebrovascular disease, acute myocardial infarction and ischaemic stroke, specifically focussed on the differential effects of R227W inclusion/exclusion. G: The effect of the R227W variant on vascular disease where controls are restricted to predicted damaging missense variants in HTRA1 (MAF<0.0001, REVEL>0.5). OR=Odds ratio CI= Confidence Interval. Test statistics plotted and P-values in C-G were derived from a generalised linear model.

To further explore the nature of the association between HTRA1 and vascular disease we reviewed variant-level associations with our coronary artery disease and cerebrovascular disease phenotypes. We were struck by the fact that the most common variant included in our gene-burden testing (HTRA1 p.R227W, hereafter R227W, N(Carriers)=386) exhibited a robust association with coronary artery disease but did not affect cerebrovascular disease risk (Figure 4A-B). This is consistent with previous work reporting that R227W had a significantly weaker effect on stroke than other curated pathogenic HTRA1 variants^29^. Follow-up analyses demonstrated that the gene-level association of HTRA1 with coronary artery disease was driven in large part by R227W, with evidence of significant heterogeneity between variant level estimates for R227W and the rest of the predicted damaging missense variants (Cochrane’s test of Heterogeneity, P(Het)=0.03). Conversely, the effect of predicted damaging missense variants in HTRA1 and cerebrovascular disease was strengthened when R227W was excluded (Figure 4C-D, Table S11). To increase our certainty in the clinical significance of these findings we restricted our phenotype definitions to clinically coded diagnoses of acute myocardial infarction and ischaemic stroke. We observed similar divergent associations between outcomes and evidence of significant heterogeneity for the myocardial infarction phenotype (Cochrane’s test of Heterogeneity, P(Het)=0.003) (Figure 4E-F, Table S11). Consistent with distinct effects of R227W in the coronary circulation carriers of R227W had a significantly increased risk of coronary artery disease (OR(CAD)=1.46, 95% CI:1.00-2.13, P=0.049) and acute myocardial infarction (OR(MI)=2.32, 95% CI=1.30-4.19, P=0.005) compared to other carriers of predicted damaging missense variants in HTRA1 (MAF<0.001, REVEL>0.5), but reduced risk of ischaemic stroke (OR(Ischaemic Stroke)=0.43, 95%CI=0.18-0.93, P=0.04) (Figure 4G).

Recently, a report was published describing inverse associations between HTRA1 activity (based on an *in vitro* assay of HTRA1-protease activity to LTBP1) and risks of stroke and coronary artery disease^40^. However, in our analyses, the association between LTBP-1 protease activity and coronary artery disease and myocardial infarction was dependent on inclusion of R227W, whereas the association with ischaemic stroke was not. While we replicated the relationship between HTRA1-protease activity and ischaemic stroke, the relationship with HTRA1-protease activity and coronary artery disease or acute MI was dependent on the inclusion of R227W (Figure S1A-D, Table S12).

### Substrate-specific impairment of HTRA1 proteolysis by the coronary artery disease risk raising variant R227W

**Figure 5:**
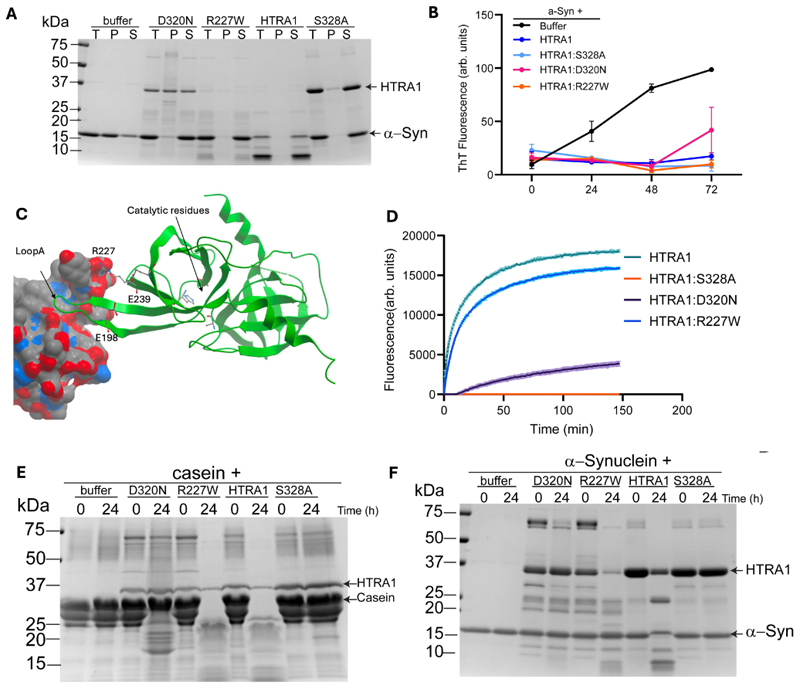
Substrate specific impairment of proteolysis by R227W HTRA1. A-B: α-synuclein sedimentation (A) and inhibition assays (B). α-synuclein monomer (25μM) was incubated in buffer, with and without the indicated HTRA variants (5μM) for 72h at 37°C with agitation of 1500 rpm in a thermomixer. For sedimentation assays, samples were collected at 72h and centrifuged to separate soluble (S) and pellet (P) fractions and analysed by SDS Page. For inhibition assays samples were mixed with ThioflavinT (ThT) dye to quantify α-synuclein aggregation. Fluorescence at 482nm was measured after excitation at 450nm using a Tecan Spark plate reader. C: Illustration of CryoEM Structure of HTRA1 bound to inhibitory fragment antibody^44^ (7SJO, only one monomer is shown) R227 binds to Fab and cannot interact with E198 and E239. D: Ability of HTRA1 mutants to cleave FITC-labelled Casein, with fluorescence indicative of casein proteolysis. E-F: Proteolysis assays of Casein 40μM (E) or α-synuclein (25μM) (F), were incubated with HTRA1 mutants for 24h at 37°C. Samples were collected at 0h and 24h and then processed by SDS-PAGE. In B, the average and SEM of three independent experiments is plotted, in A,D-F, representative results of three independent experiments are displayed.

The heterogeneous effects of coding variants in different vascular beds suggests HTRA1 may mediate vascular disease in distinct vascular beds via different mechanisms. In addition to its well described proteolytic activity, the HTRA1 proteolytic domain also confers a chaperone and disaggregase function which can prevent and reverse toxic aggregation of misfolded proteins^41–43^. Therefore, we wondered if this mechanism might be differentially affected by R227W and other variants conferring risk of cerebrovascular disease. To this end, we tested the ability of HTRA1 mutants to modulate aggregation of the model substrate alpha-synuclein. In our assessment we compared R227W with D320N, the individual variant in the protease domain most strongly associated with cerebrovascular disease with >5 carriers (OR(Cerebrovascular disease): 4.36, 95% CI: 1.78,10.66, P=4.5x10^-4^, OR(Coronary artery disease):0.73, 95% CI:0.22,2.42, P=0.58, N(carriers)=40). We purified the HTRA1 variants and alpha-synuclein and performed a sedimentation assay (Figure 5A). In the absence of HTRA1, alpha-synuclein is found largely in the insoluble fraction. However, both HTRA1 and the proteolysis-inactive HTRA1:S328A variant preserve the solubility of alpha-synuclein. Similarly, both D320N and R227W were able to prevent aggregation of alpha-synuclein. We confirmed this activity using the amyloid binding dye ThioflavinT (ThT) and again found that all of the HTRA1 variants tested prevented alpha-synuclein aggregation, indicating that this function of HTRA1 is unlikely to be relevant to the pathophysiology of HTRA1-related vascular disease (Figure 5B).

To further probe the mechanistic basis of the observed phenotypic heterogeneity, we reviewed existing structures of HTRA1. An allosteric loop A controls the conformation of the three activation loops defining the substrate binding site (Figure 5C^44^). Its productive conformation is held together by ionic interactions with E198 and E239 with R227. A therapeutic monoclonal antibody created to treat age-related macular degeneration renders HTRA1 inactive by locking the loop A and R227 in separate positions. This suggests the crucial, though indirect importance of R227 in defining the substrate binding site. Therefore, we wondered if missense variants in HTRA1 could alter proteolysis efficiency in a substrate specific manner and characterised the ability of HTRA1 mutants to cleave the model substrates casein and alpha-synuclein. Despite the effects of R227W on coronary artery disease, its ability to cleave casein was not significantly impaired, whereas D320N was severely impaired (Figure 5D-E). In contrast, the ability of R227W to cleave alpha-synuclein was markedly impaired, as was that of D320N (Figure 5F). Moreover, we observed robust auto-proteolysis of HTRA1 by R227W, but not by D320N, further evidence of the functional heterogeneity of these variants. This data establishes the precedent that R227W selectively impairs proteolysis of some substrates, providing a potential mechanistic explanation for its varying effects on coronary artery and cerebrovascular disease.

## Discussion

ASCVD remains a major cause of morbidity and mortality worldwide. Existing preventative strategies focus on risk factor modification, but increasingly there is recognition that atherosclerotic vascular events occur in the absence of standard modifiable risk factors, highlighting the need for novel therapeutic and preventative paradigms^45^. Using human genetics, we identified 3 novel candidate genes where loss of function variants raise ASCVD risk, likely independent of traditional vascular risk factors. For one of these genes, HTRA1, we provide evidence of heterogenous functional and phenotypic effects of missense variants which have important implications for our understanding of the mechanistic basis of HTRA1-related vascular disease.

HTRA1 is a homotrimeric protease that has been implicated in vascular health since its discovery as the causal gene for CARASIL (Cerebral autosomal recessive arteriopathy with subcortical infarcts and leukoencephalopathy)^28^, a cerebrovascular arteriopathy caused by bi-allelic loss of function variants in HTRA1. Population genetics efforts have demonstrated a role for heterozygous carriage of damaging HTRA1 variants in ischaemic stroke^29^ and more recently coronary artery disease^40^, but the cellular and molecular pathophysiology of HTRA1-related vascular disease remains poorly understood. Our demonstration of functional and phenotypic heterogeneity resulting from missense variants in HTRA1 is consistent with distinct pathophysiological processes driving vascular disease in the cerebrovascular and coronary circulations. This appears consistent with anecdotal evidence from a histopathological case series that studied the cerebral and systemic vasculature of patients with CARASIL^46^. Characteristic cerebral arteriopathy was observed with loss of medial vascular smooth muscle cells. However, in coronary arteries medial vascular smooth muscle cells appeared preserved, but pathological neointimal thickening was observed in the absence of significant atheromatous plaque^46^.

Several studies have suggested that impairments in HTRA1 protease activity drive HTRA1-related vascular disease^26,28,31,40,47,48^. In particular, a recent study characterised 78 missense variants in HTRA1 present in population biobanks and found an association between the capacity of mutant HTRA1 activity to proteolyse LTBP1 and risk of both coronary and cerebrovascular disease^40^. Our findings, that the R227W variant drives coronary artery disease risk in UK Biobank and that this variant exhibits impairment to a subset of tested HTRA1 substrates, suggests a more nuanced relationship between HTRA1 proteolytic capacity and vascular risk. Specifically, we suggest that the risk of coronary artery disease conferred by the R227W variant relates to a modified substrate repertoire, which is reflected in its inability to cleave certain substrates. Identifying this set of substrates will provide key insight into the molecular pathophysiology of HTRA1-related vascular disease. Importantly, this model does not imply that damaging variants in HTRA1 cannot cause both clinically manifest cerebrovascular and coronary artery disease - variants which have no proteolytic capacity to any substrate or change expression of HTRA1 would be expected to alter risk of both pathologies. Consistent with this, common non-coding variants in HTRA1 which likely affect vascular risk by changing vascular HTRA1 expression increase risk of both cerebrovascular and coronary artery disease.

While HTRA1 is best known for its proteolytic capacity it has other molecular functions including chaperone activity and disaggregase function^41,43,49^. Interestingly, both D320N and R227W HTRA1 variants retained inhibitory activity, preventing alpha-synuclein aggregation, suggesting that HTRA1 chaperone activity is not required for maintenance of vascular health.

We identified two other genes, SGTB and RBM12, where loss of function variants are associated with an increased risk of ACVSD. RBM12 is an RNA-binding protein that has been reported to bind to the 5’ region of mRNA and regulate transcript splicing, mRNA localisation and translation^35,50,51^. Moreover, unbiased screens have identified RBM12 as a repressor of GPCR-cAMP dependent signalling^52^. Loss of function variants in RBM12 have not previously been linked to vascular risk. A previous study has reported PTVs in RBM12 to segregate with psychosis in an Icelandic and Finnish cohort but did not comment on ASCVD disease in this pedigree^53^. The molecular function of SGTB is poorly characterised, but its homologue SGTA has a well described role as a molecular chaperone^33^. Our work provides impetus to elucidate the role of these proteins in vascular cells and to study their impact on atherosclerosis in model systems to provide orthogonal validation of these hypothesis generating findings.

Our rationale for aggregating atherosclerotic cardiovascular disease across multiple vascular beds as a phenotype was two-fold: i) similar pathophysiological processes contribute to its development and thus power to detect genes driving common processes across vascular beds may be increased ii) current paradigms for assessing efficacy of prevention and treatment of vascular disease typically assess capacity of interventions to reduce vascular risk across at least two vascular beds (typically coronary artery disease and cerebrovascular disease as part of Major Adverse Cardiovascular Events endpoint) so genes which increase risk across multiple vascular beds may be more attractive therapeutic targets. This approach was successful in identification of 3 genes which appear to increase risk of vascular disease independent of established vascular risk factors, and two traits where the amalgamation of phenotypes resulted in Exome-wide significant associations which would have not been discovered if a single trait had been explored. However, it should be noted that there was evidence of heterogeneity of effects across vascular beds at a gene-burden test level and, as highlighted, at the variant level for HTRA1. Future studies could consider adopting analytical approaches that can account for this observed heterogeneity to maximise statistical power.

In summary, we have used a population genetics approach to identify novel genetic risk factors for atherosclerotic cardiovascular disease. We have provided evidence of heterogenous effects of HTRA1 variants across vascular beds with concordant functional heterogeneity suggesting a distinct mechanistic basis for HTRA1-related vascular disease in the coronary and cerebrovasculature.

## Supplementary Material

005233 - Supplemental Material

005233 - Supplemental Tables
