# Rare Variants in HTRA1, SGTB, and RBM12 Confer Risk of Atherosclerotic Cardiovascular Disease Independent of Traditional Cardiovascular Risk Factors

**DOI:** 10.1161/CIRCGEN.125.005233

**Published:** 2025-11-05

**Authors:** Sam M. Lockhart, Anuradhika Puri, Yajie Zhao, Vladimir Saudek, Eugene J. Gardner, Katherine A. Kentistou, Brian Y.H. Lam, Felix R. Day, Stephen O’Rahilly, John R.B. Perry, Ken K. Ong, Meredith E. Jackrel

**Affiliations:** 1 Wellcome-Wolfson Institute for Experimental Medicine, Queen’s University Belfast, United Kingdom (S.M.L.).; 2 Metabolic Research Laboratories and NIHR Cambridge Biomedical Research Centre, Institute of Metabolic Science (S.M.L., V.S., S.O., B.Y.H.L.), University of Cambridge, United Kingdom.; 3 Department of Paediatrics (K.K.O.), University of Cambridge, United Kingdom.; 4 MRC Epidemiology Unit, Institute of Metabolic Science (Y.Z., E.J.G., K.A.K., F.R.D., J.R.B.P., K.K.O., J.R.B.P.), University of Cambridge, United Kingdom.; 5 Department of Paediatrics (K.K.O.), University of Cambridge, United Kingdom.; 6 Department of Chemistry, Washington University, St. Louis, MO (A.P., M.E.J.).

**Keywords:** atherosclerosis, cardiovascular diseases, myocardial ischemia, stroke

## Abstract

**BACKGROUND:**

Atherosclerosis is a pathophysiological process common to a range of cardiovascular diseases. We reasoned that considering clinical presentations of atherosclerosis across the coronary, peripheral, and cerebrovasculature as a single entity would enhance statistical power to identify rare genetic variation driving pathological processes across multiple vascular beds.

**METHODS:**

We performed an exome-wide association study of atherosclerotic cardiovascular disease in 434 438 UK Biobank participants of European ancestry.

**RESULTS:**

We identified rare, predicted damaging variants in HTRA1, SGTB, and RBM12 to be associated with risk of atherosclerotic cardiovascular disease, independent of known risk factors. Both SGTB and HTRA1 were downregulated in the aorta of patients with coronary artery disease compared with controls. Loss-of-function variants in the RNA-binding protein RBM12 increased the risk of coronary, cerebrovascular, and peripheral vascular diseases to a similar extent. SGTB increased the risk of atherosclerotic cardiovascular disease in the coronary and peripheral circulations but not the cerebrovasculature. While loss-of-function variants in HTRA1 are known to cause monogenic stroke syndromes, we found that damaging missense variants in HTRA1 are associated with increased risk of disease in both the cerebrovascular and coronary circulation. Surprisingly, the increased risk of coronary artery disease was driven predominantly by a single missense variant (p.R227W; minor allele frequency, 0.009). In vitro, the R227W mutant HTRA1 efficiently proteolyzed the disordered substrate casein but not aggregated α-synuclein. In contrast, a stroke risk–raising variant (D320N) could not efficiently process any of the tested substrates.

**CONCLUSIONS:**

We identified novel genetic variants predisposing to atherosclerotic cardiovascular diseases that act independently of established cardiovascular risk factors. The observed phenotypic and functional heterogeneities between HTRA1 variants suggest that distinct biochemical mechanisms drive HTRA1-related vascular disease in the brain and heart.

Atherosclerosis causes ischemic vascular disease in the brain, heart, and peripheral vasculature. The development of efficacious prevention strategies targeting cardiovascular risk factors such as hypertension, LDL cholesterol, and other ApoB (apolipoprotein-B) containing lipoproteins has revolutionized the care of these conditions. However, stroke and ischemic heart disease remain the leading causes of morbidity and mortality in adults worldwide,^1^ and the morbidity attributable to peripheral vascular disease is increasing.^2–4^ Recent estimates suggest that >40% of cardiovascular disease occurs independently of traditional cardiovascular risk factors.^5^ These data, combined with increasing recognition of ischemic cardiovascular events occurring in the absence of standard modifiable risk factors,^6^ provide renewed impetus to discover novel drivers of atherosclerotic cardiovascular disease (ASCVD).^7,8^

Human genetics is unique in its capacity to identify novel causal drivers of disease in an unbiased fashion and is increasingly used to prioritize therapeutic targets^9^ for drug development. Common variant association studies support biological processes acting directly in the vascular wall as modifiers of cardiovascular risk.^10–12^ However, uncertainty around the annotation of causal variants and genes means that translating these associations to a mechanistic understanding of disease is challenging.^13^ In contrast, assessment of rare coding variation using exome-wide sequencing data can confidently annotate a causal gene.^14–17^ This approach has provided insights into a range of cardiometabolic diseases, including coronary artery disease, where exome-sequencing–based studies have identified low-frequency variants associated with coronary artery disease risk (10%<minor allele frequency <1%).^18,19^ However, systematic examination for rare coding variation associated with ASCVD has met with limited success, potentially due to a lack of statistical power.^20^

Traditionally, genetic determinants of ASCVDs have been studied separately. However, they share a fundamental pathological process and are correlated genetically and observationally. We reasoned that the amalgamation of ASCVD across multiple vascular beds would enhance statistical power to identify genes with a causal role in vascular disease.

To this end, we performed an exome-wide association study of ASCVD in the UK Biobank (UKBB), identifying 3 novel ASCVD risk genes acting independently of traditional cardiovascular risk factors. For one such gene, *HTRA1*, we find evidence of functional and phenotypic heterogeneities with distinct missense variants raising coronary and cerebrovascular risk. Together, our findings provide novel insight into the pathophysiology of ASCVD and vascular multimorbidity.

## Methods

### Data Availability

Rare variant association testing described in this article was conducted using the MRC Epidemiology Unit, Cambridge Whole exome sequencing pipelines (https://github.com/mrcepid-rap/). All data used in the genetic association analyses are available from the UKBB upon application (https://www.ukbiobank.ac.uk).

### Ethical Approval

The UKBB data have approval from the North West Multi-Center Research Ethics Committee as a research tissue bank. All participants provided written informed consent. This research has been conducted using the UKBB Resource under application number 9905. All analyses reported here were conducted in accordance with relevant ethical guidelines.

Further information regarding methods is provided in the Supplemental Material.

## Results

### An Exome-Wide Association Study of ASCVD Implicates HTRA1, Small Glutamine-Rich Tetratricopeptide Repeat Co-Chaperone Beta, and RBM12 in Atherosclerosis Across Multiple Vascular Beds

To identify novel genetic drivers of ASCVD, we conducted an exome-wide association study for ASCVD in 434 438 UKBB participants of European ancestry. ASCVD cases were defined as any participant with evidence of coronary, cerebrovascular, or peripheral vascular disease (n=61 598; case prevalence, 14.2%; Table S1; Supplemental Methods). Gene-based collapsing tests were performed by aggregating rare (minor allele frequency <0.001) variants across 19 457 protein-coding genes as described previously.^16,17^ Burden testing was conducted using 3 variant masks per gene: predicted loss-of-function protein-truncating variants (PTVs) as defined by loss-of-function transcript effect estimator^21^ and predicted damaging missense variants with Rare Exome Variant Ensemble Learner scores >0.5 or >0.7.^22^ This resulted in a total of 37 686 individual gene×mask combinations and a stringent, Bonferroni-corrected multiple testing threshold of *P*<1.33×10^−6^. Our results appeared statistically well calibrated as evidenced by the absence of any synonymous variant mask associations with ASCVD (included as a negative control) and low exome-wide test-statistic inflation (λ_GC_=1.03).

**Figure 1.**
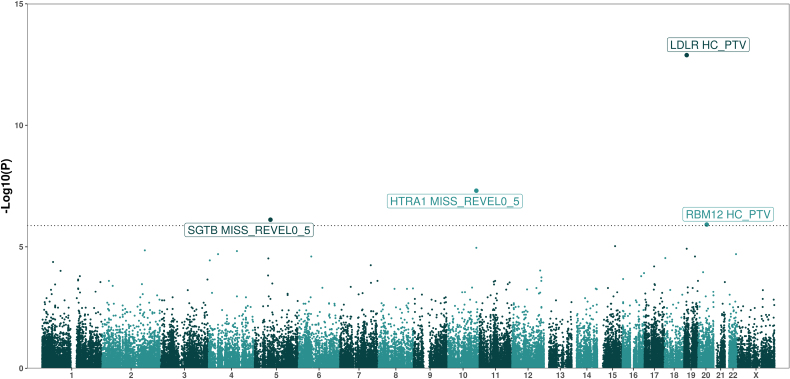
**An exome-wide association study of atherosclerotic cardiovascular disease in UK Biobank (UKBB) participants of European ancestry.** Manhattan plot summarizing an exome-wide association study of atherosclerotic cardiovascular disease in UKBB. Each dot represents an individual gene×mask combination. The dotted line represents a Bonferroni-corrected multiple testing threshold of 1.33×10-6. The labels represent gene names where any single gene×mask combination is significantly associated with atherosclerotic cardiovascular disease. Test statistics were derived from a linear mixed model of European participants in UKBB (implemented in BOLT-LMM, Bayesian, Orthogonal, at Large-scale, Linear Mixed Model) with adjustment for age, age-squared, sex, the first 10 genetic principal components, and whole exome–sequencing batch.

We identified 4 unique genes associated with ASCVD: LDLR, HTRA1, SGTB, and RBM12 (Figure 1; Table S2). Associations with ASCVD were not sensitive to variations in the basis of phenotype definitions (eg, inclusion/exclusion of self-reported cases), and leave-one-out analyses did not suggest that any association was driven solely by a single variant (Tables S3 and S4). We observed similar associations using an orthogonal statistical modeling framework (linear mixed models implemented in the scalable and accurate implementation of generalized mixed model-gene-based association test) though *P* values were somewhat attenuated compared with those obtained in discovery with BOLT-LMM (Table S14).

**Figure 2.**
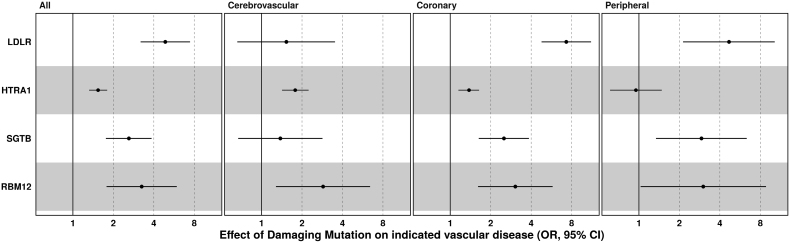
**Atherosclerotic cardiovascular disease risk genes increase disease risk via actions in multiple vascular beds.** To provide insight into the tissue of action of the candidate genes, we partitioned our atherosclerotic cardiovascular disease phenotype into vascular beds and assessed the effect of the most strongly associated mask for each of the 3 exome-wide significant atherosclerotic cardiovascular risk genes. The test statistics plotted are from a generalized linear model after adjustment for the first 10 genetic principal components, age, age-squared, and sex. *P* values from collapsing burden tests implemented in BOLT-LMM (Bayesian, Orthogonal, at Large-scale, Linear Mixed Model) are available in Table S5. LDLR indicates low-density lipoprotein receptor; OR, odds ratio; and SGTB, small glutamine-rich tetratricopeptide repeat co-chaperone beta.

LDLR encodes the low-density lipoprotein receptor. Monoallelic or biallelic loss-of-function variants in LDLR cause familial hypercholesterolemia and premature onset of ASCVD. Carriers of PTVs in LDLR exhibited an almost 5-fold increase in odds of ASCVD (OR, 4.87 [95% CI, 3.19–7.44]; *P*=1.30×10^−13^; carriers=75; case prevalence=37%) that was driven predominantly by disease in the peripheral and coronary circulation (Figure 2; Table S5), consistent with existing data on monogenic familial hypercholesterolemia.^23–25^

HTRA1 is a homotrimeric serine protease that is expressed in endothelial and smooth muscle cells of the vasculature.^26–28^ Biallelic loss-of-function variants cause arteriopathy in the cerebral circulation, which presents clinically with premature stroke and early-onset vascular dementia.^28^ Rare, predicted damaging missense variants (Rare Exome Variant Ensemble Learner score >0.5) were associated with ≈50% increase in the odds of ASCVD (OR, 1.54 [95% CI, 1.32–1.80]; *P*=4.9×10^−8^). It is well-established that heterozygous carriage of loss-of-function variants in HTRA1 increases risk of ischemic stroke and imaging biomarkers of cerebrovascular disease.^29–31^ However, this association was driven by both an increased risk of disease in both the coronary (OR, 1.38 [95% CI, 1.15–1.64]; *P*=5.4×10^−4^) and cerebrovasculature (OR, 1.78 [95% CI, 1.42–2.24]; *P*=5.50×10^−7^; Figure 2; Table S5).

SGTB is a poorly characterized protein that is broadly expressed but exhibits particular enrichment in the brain. Its homologue, SGTA, has a role in regulating localization and disposal of misfolded proteins,^32–34^ but SGTB has not been well studied in any biological context. It is likely that, like SGTA, the N-terminal domain of SGTB facilitates formation of homodimers. It then functions in a chaperone complex where its C-terminal domain recognizes and facilitates interaction with client proteins, modulating their localization and degradation. Missense variants (Rare Exome Variant Ensemble Learner score >0.5) in SGTB appeared to raise risk of ASCVD primarily via effects in the coronary and peripheral circulation (Figure 2; OR (coronary), 2.51 [95% CI, 1.63–3.85]; *P*=1.5×10^−5^; OR (peripheral), 2.92 [95% CI, 1.34–6.34]; *P*=4.8×10^−3^). Interestingly, the effects of SGTB appeared to be driven by 2 distinct missense variants (p.SGTB A138V and A228V; OR (ASCVD), 2.84 [95% CI, 1.83–4.39]; *P*=2.9×10^−6^), one in the highly conserved tetratricopeptide repeat domain and one in the C-terminal domain, which recognizes client proteins. While the alanine to valine substitution is relatively mild, both of these residues are extremely well conserved (Figure S1), predicted to be damaging by multiple orthogonal bioinformatic predictors, and occur in structurally important regions buried inside the tightly packed complex. This supports the deleteriousness of these variants and the assertion that damaging variants in SGTB increase ASCVD risk.

Finally, RBM12 is a poorly characterized RNA-binding protein likely implicated in the regulation of premRNA splicing.^35^ PTVs in RBM12 appeared to increase the risk of vascular disease across all 3 subphenotypes studied with ≈3-fold increase in odds of coronary, cerebrovascular, and peripheral vascular diseases (Figure 2; Table S5).

### Damaging Variants in HTRA1, SGTB, and RBM12 Are Not Associated With Changes in Traditional Cardiovascular Risk Factors

**Figure 3.**
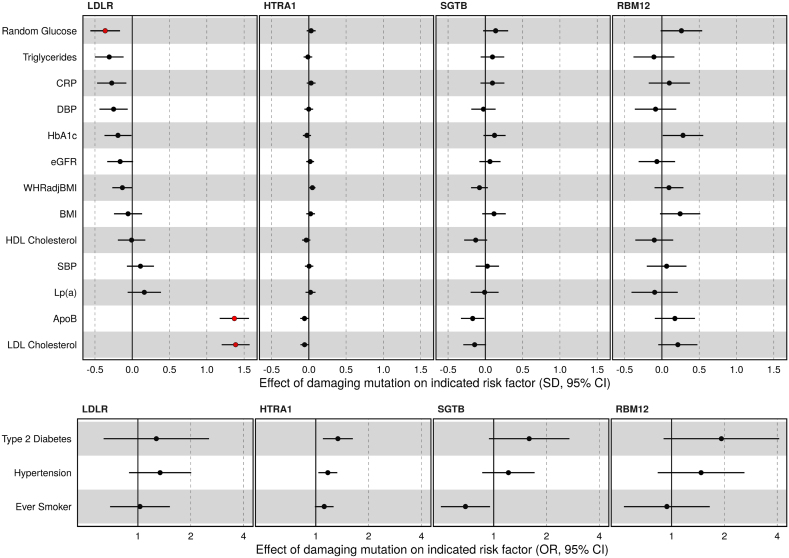
**Effects of pLOF variants in atherosclerotic cardiovascular disease risk genes on selected cardiovascular risk factors.** The effects of the atherosclerotic risk genes on continuous (**top**) and dichotomized cardiovascular risk factors (**bottom**) are shown. For each gene, the variant mask most significantly associated with atherosclerotic cardiovascular disease is plotted. The points coloured red are significantly associated with the indicated risk factor after adjustment for multiple testing. ApoB indicates apolipoprotein-B; BMI, body mass index; OR, odds ratio; SBP, systolic blood pressure; CRP, c-reactive protein; DBP, diastolic blood pressure; HbA1c, Haemoglobin A1c; eGFR, estimated glomerular filtration rate; WHR, waist-hip ratio; HDL, High density lipoprotein; Lp(a), Lipoprotein(a); LDL, low density lipoprotein; pLOF, predicted loss of function.

Atherosclerosis has many well-established causal risk factors, which could be driving the effect of identified risk genes. To determine if this was the case, we assessed the effect of ASCVD risk genes on a panel of cardiovascular risk factors. As expected, PTVs in LDLR had a large effect on LDL cholesterol and ApoB (Table S7; Figure 3). Consistent with known effects of chronic elevations in LDL cholesterol on glycemia,^36,37^ random glucose was significantly reduced in carriers of LDLR PTVs though there was no association with type 2 diabetes risk or HbA1c, after adjustment for multiple testing (Tables S7 and S8; Figure 3). In contrast, there was no significant association with predicted damaging variants in HTRA1, RBM12, or SGTB with any of the tested cardiovascular risk factors, after adjustment for multiple testing. Consistent with effects of damaging variants in HTRA1, SGTB, and RBM12 acting independently of traditional cardiovascular risk factors, these associations persisted after adjusting for LDL cholesterol, smoking, type 2 diabetes, body mass index, and systolic blood pressure (Table S9).

### HTRA1 and SGTB Are Robustly Expressed in Vascular Tissue and Are Downregulated in Patients With Coronary Artery Disease

Given the lack of a convincing association between the novel ASCVD risk genes and established cardiovascular risk factors, we reasoned that these genes could be mediating their effects via direct actions in the vasculature. To assess the biological plausibility of this hypothesis, we examined the expression distribution of these genes in the Stockholm-Tartu Atherosclerosis Reverse Network Engineering Task database, which contains transcriptomic data from patients undergoing coronary artery bypass grafting and controls.^38^ Consistent with the known biology of HTRA1, it was robustly expressed in the aorta and mammary artery (aorta: median expression [Q1, Q3]=385 [308, 475] cpm; internal mammary artery: median expression [Q1, Q3]=241 [206, 280] cpm). Interestingly, HTRA1 expression was significantly downregulated in the aorta from patients with atherosclerotic coronary artery disease versus controls (false discovery rate–corrected *P*=3.4×10^−4^). In contrast to the well-described direct actions of HTRA1 in the cerebral vasculature, the biological significance of RBM12 and SGTB in the vascular wall is unknown. Both RBM12 (aorta: median expression [Q1, Q3]=123 [110, 135] cpm; internal mammary artery: median expression [Q1, Q3]=126 [113, 139] cpm) and SGTB (aorta: median expression [Q1, Q3]=70 [64, 78] cpm; internal mammary artery: median expression [Q1, Q3]=74 [66, 83] cpm) were robustly expressed in aorta and internal mammary artery. While RBM12 was not differentially regulated in the aorta from patients with cardiovascular disease, SGTB was significantly downregulated (false discovery rate–corrected *P*=2.1×10^−5^). These data are consistent with reduced expression of HTRA1 and SGTB contributing to atherosclerosis in sporadic ASCVD.

### Predisposition to Coronary Artery Disease Is Not a General Feature of Monogenic Cerebral Small Vessel Disease

The association of coronary artery disease with predicted damaging variants in HTRA1 (Figure 2) led us to consider whether disease of the coronary circulation might be an unrecognized feature of other monogenic cerebral small vessel disease syndromes. Cerebral autosomal dominant arteriopathy with subcortical infarcts and leukoencephalopathy is an autosomal dominant cerebral small vessel disease syndrome caused by missense variants in NOTCH3, and it shares some of the clinical and pathophysiological features of HTRA1-related cerebrovascular disease,^39^ including early-onset stroke and vascular dementia. We examined the relationship between pathogenic (eg, cerebral autosomal dominant arteriopathy with subcortical infarcts and leukoencephalopathy-causing) variants in NOTCH3^29^ and coronary artery disease. As expected, pathogenic variants were associated with an increased risk of cerebrovascular disease (OR, 2.14 [95% CI, 1.68–2.74]; *P*=4.43×10^−10^; Table S10). However, there was no increased risk of coronary disease (OR, 0.79 [95% CI, 0.60–1.03]; *P*=0.08). Thus, the effect of HTRA1 on the coronary vascular health is not a general feature of cerebral small vessel disease and may be related to specific effects of HTRA1 in the coronary vasculature.

### Distinct Variants Drive HTRA1-Related Vascular Disease in the Cerebral and Coronary Circulations

**Figure 4.**
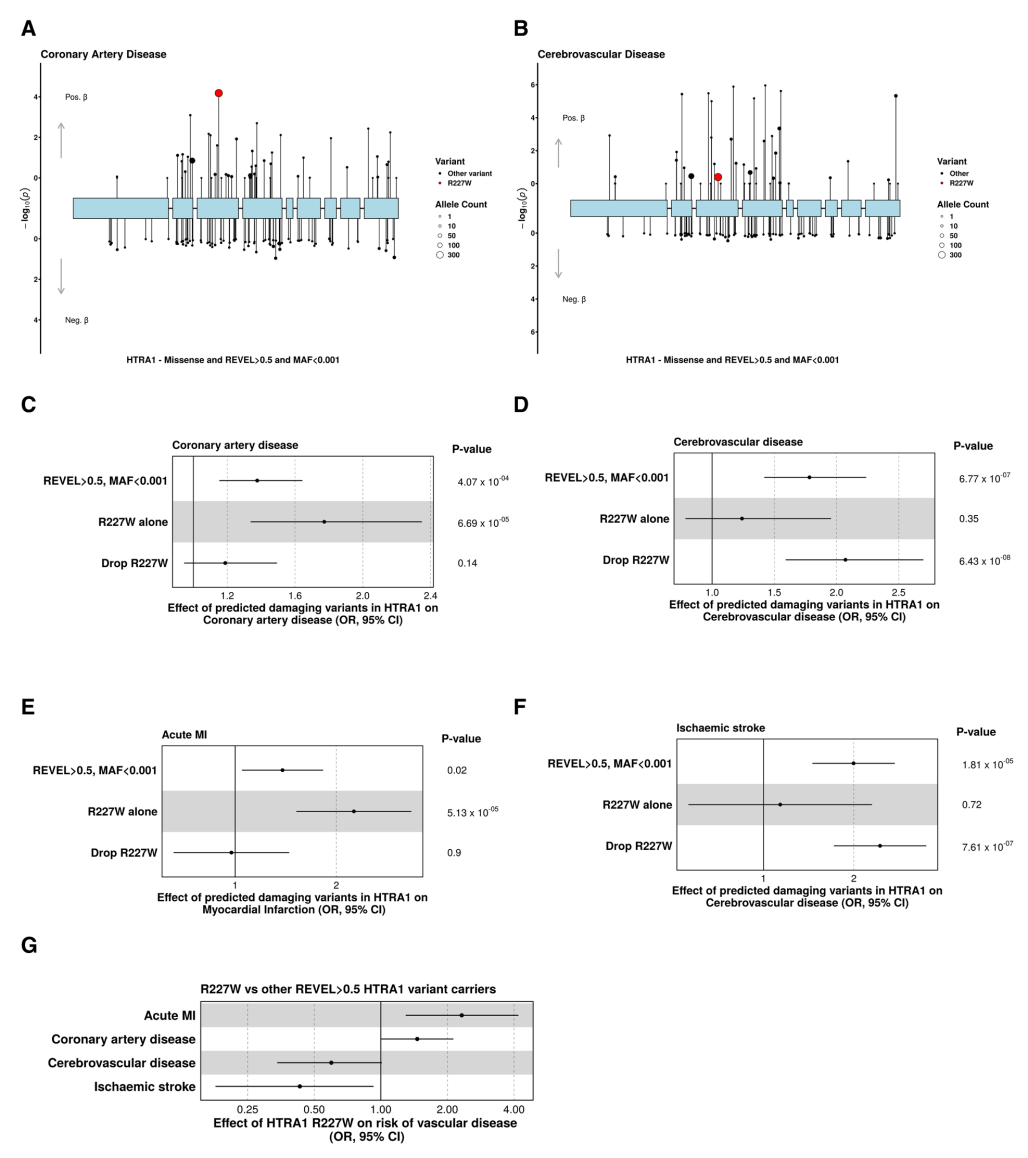
**p.R227W increases coronary artery disease risk but not risk of cerebrovascular disease.** Lolliplots of variants in HTRA1 and their effect on coronary artery disease (**A**) and cerebrovascular disease risk (**B**). The size of each dot is proportional to the number of carriers. The red dot represents p.R227W, which has differential effects on cerebrovascular and coronary artery disease. Test statistics plotted were derived from a linear mixed model implemented in BOLT. **C** through **F**, Effect of mask composition on coronary artery disease, cerebrovascular disease, acute myocardial infarction, and ischemic stroke, specifically focused on the differential effects of R227W inclusion/exclusion. **G**, The effect of the R227W variant on vascular disease, where controls are restricted to predicted damaging missense variants in HTRA1 (minor allele frequency [MAF] <0.0001; Rare Exome Variant Ensemble Learner [REVEL] >0.5). Test statistics plotted and *P* values in **C** through **G** were derived from a generalized linear model. MI indicates myocardial infarction; and OR, odds ratio.

To further explore the nature of the association between HTRA1 and vascular disease, we reviewed variant-level associations with our coronary artery disease and cerebrovascular disease phenotypes. We were struck by the fact that the most common variant included in our gene-burden testing (HTRA1 p.R227W, hereafter R227W, n [carriers]=386) exhibited a robust association with coronary artery disease but did not affect cerebrovascular disease risk (Figure 4A and 4B). This is consistent with previous work reporting that R227W had a significantly weaker effect on stroke than other curated pathogenic HTRA1 variants.^29^ Follow-up analyses demonstrated that the gene-level association of HTRA1 with coronary artery disease was driven in large part by R227W, with evidence of significant heterogeneity between variant-level estimates for R227W and the rest of the predicted damaging missense variants (Cochrane test of heterogeneity, *P*_Het_=0.03). Conversely, the effect of predicted damaging missense variants in HTRA1 and cerebrovascular disease was strengthened when R227W was excluded (Figure 4C and 4D; Table S11). To increase our certainty in the clinical significance of these findings, we restricted our phenotype definitions to clinically coded diagnoses of acute myocardial infarction (MI) and ischemic stroke. We observed similar divergent associations between outcomes and evidence of significant heterogeneity for the MI phenotype (Cochrane test of heterogeneity, *P*_Het_=0.003; Figure 4E and 4F; Table S11). Consistent with distinct effects of R227W in the coronary circulation versus the cerebrovasculature: carriers of R227W had a significantly increased risk of coronary artery disease (OR [CAD], 1.46 [95% CI, 1.00–2.13]; *P*=0.049) and acute MI (OR [MI]; 2.32 [95% CI, 1.30–4.19]; *P*=0.005) compared with other carriers of predicted damaging missense variants in HTRA1 (minor allele frequency <0.001; Rare Exome Variant Ensemble Learner score >0.5) but reduced risk of ischemic stroke (OR [ischemic stroke], 0.43 [95% CI, 0.18–0.93]; *P*=0.04; Figure 4G).

Recently, a report was published describing inverse associations between HTRA1 activity (based on an in vitro assay of HTRA1-protease activity to LTBP1 [latent transforming growth factor beta binding protein 1]) and risks of stroke and coronary artery disease.^40^ While we replicated the relationship between HTRA1-protease activity and ischemic stroke, the relationship with HTRA1-protease activity and coronary artery disease or acute MI was dependent on the inclusion of R227W (Figure S1A through S1D; Table S12).

### Substrate-Specific Impairment of HTRA1 Proteolysis by the Coronary Artery Disease Risk–Raising Variant R227W

**Figure 5.**
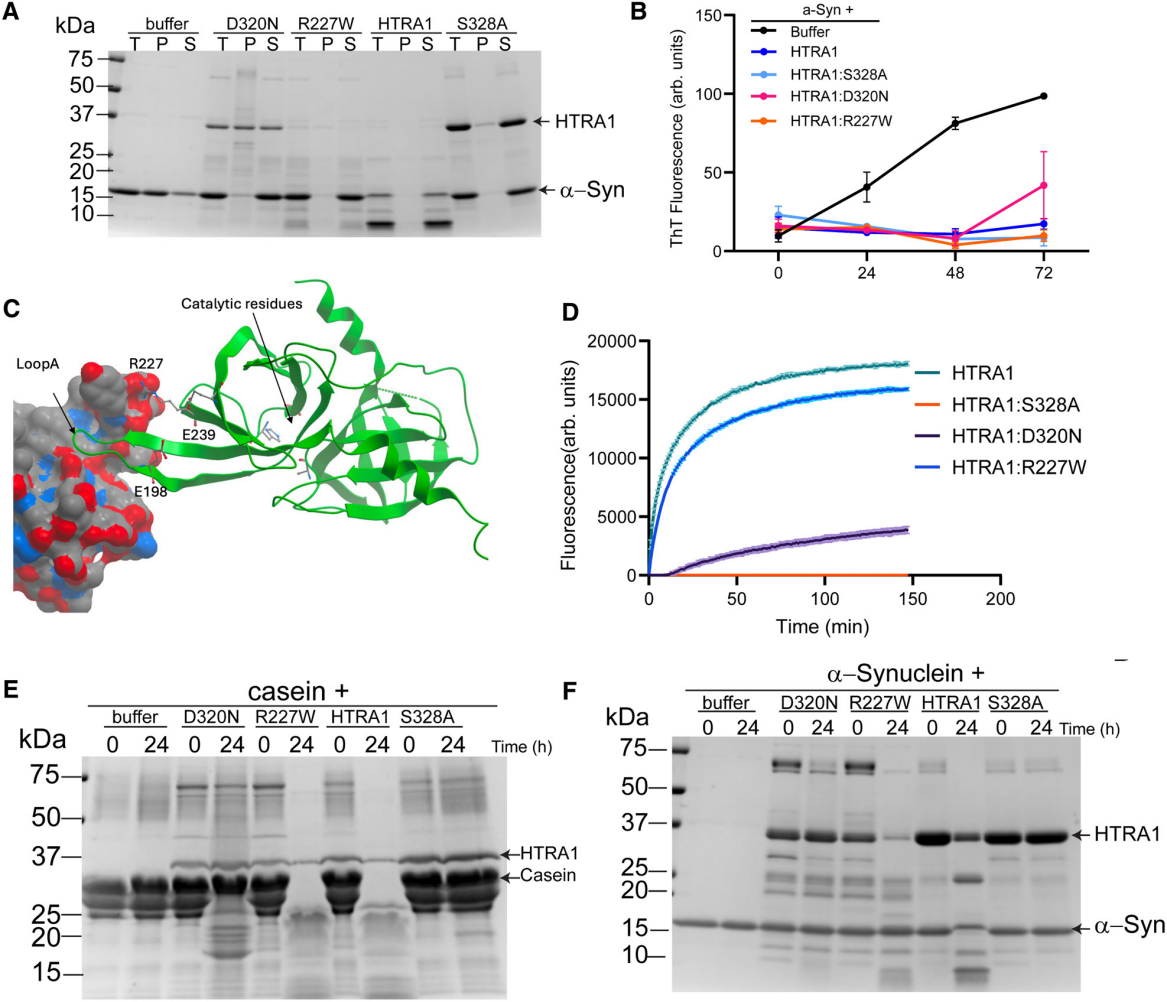
**Substrate-specific impairment of proteolysis by R227W HTRA1.** α-Synuclein sedimentation (**A**) and inhibition assays (**B**). α-Synuclein monomer (25 μM) was incubated in buffer, with and without the indicated HTRA variants (5 μM) for 72 hours at 37 °C with agitation of 1500 rpm in a thermomixer. For sedimentation assays, samples were collected at 72 hours and centrifuged to separate soluble (S) and pellet (P) fractions and analyzed by SDS-PAGE. For inhibition assays, samples were mixed with thioflavin T (ThT) dye to quantify α-synuclein aggregation. Fluorescence at 482 nm was measured after excitation at 450 nm using a Tecan Spark plate reader. **C**, Illustration of CryoEM structure of HTRA1 bound to inhibitory fragment antibody^44^ (7SJO, only 1 monomer is shown) R227 binds to Fab and cannot interact with E198 and E239. **D**, Ability of HTRA1 mutants to cleave fluorescein isothiocyanate (FITC)–labeled casein, with fluorescence indicative of casein proteolysis. Proteolysis assays of casein 40 μM (**E**) or α-synuclein (25 μM; **F**) were incubated with HTRA1 mutants for 24 hours at 37 °C. Samples were collected at 0 and 24 hours and then processed by SDS-PAGE. In **B**, the average and SEM of 3 independent experiments are plotted. In **A** and **D** through **F**, representative results of 3 independent experiments are displayed.

The heterogeneous effects of coding variants in different vascular beds suggest that HTRA1 may mediate vascular disease in distinct vascular beds via different mechanisms. In addition to its well-described proteolytic activity, the HTRA1 proteolytic domain also confers a chaperone and disaggregase function, which can prevent and reverse toxic aggregation of misfolded proteins.^41–43^ Therefore, we wondered if this mechanism might be differentially affected by R227W and other variants conferring risk of cerebrovascular disease. To this end, we tested the ability of HTRA1 mutants to modulate aggregation of the model substrate α-synuclein. In our assessment, we compared R227W with D320N, the individual variant in the protease domain most strongly associated with cerebrovascular disease with >5 carriers (OR [cerebrovascular disease], 4.36 [95% CI, 1.78–10.66]; *P*=4.5×10^−4^; OR [coronary artery disease], 0.73 [95% CI, 0.22–2.42]; *P*=0.58; N [carriers]=40). We purified the HTRA1 variants and α-synuclein and performed a sedimentation assay (Figure 5A). In the absence of HTRA1, α-synuclein is found largely in the insoluble fraction. However, both HTRA1 and the proteolysis-inactive HTRA1:S328A variant preserve the solubility of α-synuclein. Similarly, both D320N and R227W were able to prevent aggregation of α-synuclein. We confirmed this activity using the amyloid-binding dye thioflavin T and again found that all of the HTRA1 variants tested prevented α-synuclein aggregation, indicating that this function of HTRA1 is unlikely to be relevant to the pathophysiology of HTRA1-related vascular disease (Figure 5B).

To further probe the mechanistic basis of the observed phenotypic heterogeneity, we reviewed existing structures of HTRA1. An allosteric loop A controls the conformation of the 3 activation loops defining the substrate binding site (Figure 5C).^44^ Its productive conformation is held together by ionic interactions with E198 and E239 with R227. A therapeutic monoclonal antibody created to treat age-related macular degeneration renders HTRA1 inactive by locking the loop A and R227 in separate positions. This suggests the crucial, though indirect, importance of R227 in defining the substrate binding site. Therefore, we wondered if missense variants in HTRA1 could alter proteolysis efficiency in a substrate-specific manner and characterized the ability of HTRA1 mutants to cleave the model substrates casein and α-synuclein. Despite the effects of R227W on coronary artery disease, its ability to cleave casein was not significantly impaired, whereas D320N was severely impaired (Figure 5D and 5E). In contrast, the ability of R227W to cleave α-synuclein was markedly impaired, as was that of D320N (Figure 5F). Moreover, we observed robust autoproteolysis of HTRA1 by R227W, but not by D320N, further evidence of the functional heterogeneity of these variants. These data establish the precedent that R227W selectively impairs proteolysis of some substrates, providing a potential mechanistic explanation for its varying effects on coronary artery and cerebrovascular disease.

## Discussion

ASCVD remains a major cause of morbidity and mortality worldwide. Existing preventative strategies focus on risk factor modification, but, increasingly, there is recognition that atherosclerotic vascular events occur in the absence of standard modifiable risk factors, highlighting the need for novel therapeutic and preventative paradigms.^45^ Using human genetics, we identified 3 novel candidate genes where loss-of-function variants raise ASCVD risk, likely independent of traditional vascular risk factors. For one of these genes, HTRA1, we provide evidence of heterogeneous functional and phenotypic effects of missense variants, which have important implications for our understanding of the mechanistic basis of HTRA1-related vascular disease.

HTRA1 is a homotrimeric protease that has been implicated in vascular health since its discovery as the causal gene for cerebral autosomal recessive arteriopathy with subcortical infarcts and leukoencephalopathy,^28^ a cerebrovascular arteriopathy caused by biallelic loss-of-function variants in HTRA1. Population genetics efforts have demonstrated a role for heterozygous carriage of damaging HTRA1 variants in ischemic stroke^29^ and, more recently, coronary artery disease,^40^ but the cellular and molecular pathophysiology of HTRA1-related vascular disease remains poorly understood. Our demonstration of functional and phenotypic heterogeneities resulting from missense variants in HTRA1 is consistent with distinct pathophysiological processes, driving vascular disease in the cerebrovascular and coronary circulations. This appears consistent with anecdotal evidence from a histopathologic case series that studied the cerebral and systemic vasculatures of patients with cerebral autosomal recessive arteriopathy with subcortical infarcts and leukoencephalopathy.^46^ Characteristic cerebral arteriopathy was observed with loss of medial vascular smooth muscle cells. However, in coronary arteries, medial vascular smooth muscle cells appeared preserved, but pathological neointimal thickening was observed in the absence of significant atheromatous plaque.^46^

Several studies have suggested that impairments in HTRA1 protease activity drive HTRA1-related vascular disease.^26,28,31,40,47,48^ In particular, a recent study characterized 78 missense variants in HTRA1 present in population biobanks and found an association between the capacity of mutant HTRA1 activity to proteolyse LTBP1 and risk of both coronary and cerebrovascular disease.^40^ Our findings that the R227W variant drives coronary artery disease risk in UKBB and that this variant exhibits impairment to a subset of tested HTRA1 substrates suggest a more nuanced relationship between HTRA1 proteolytic capacity and vascular risk. Specifically, we suggest that the risk of coronary artery disease conferred by the R227W variant relates to a modified substrate repertoire, which is reflected in its inability to cleave certain substrates. Identifying this set of substrates will provide key insight into the molecular pathophysiology of HTRA1-related vascular disease. Importantly, this model does not imply that damaging variants in HTRA1 cannot cause both clinically manifest cerebrovascular and coronary artery disease; variants that have no proteolytic capacity to any substrate or change expression of HTRA1 would be expected to alter the risk of both pathologies. Consistent with this, common noncoding variants in HTRA1, which likely affect vascular risk by changing vascular HTRA1 expression, increase the risk of both cerebrovascular and coronary artery disease.

While HTRA1 is best known for its proteolytic capacity, it has other molecular functions, including chaperone activity and disaggregase function.^41–43^ Interestingly, both D320N and R227W HTRA1 variants retained inhibitory activity, preventing α-synuclein aggregation, suggesting that HTRA1 chaperone activity is not required for maintenance of vascular health.

We identified 2 other genes, *SGTB* and *RBM12*, where loss-of-function variants are associated with an increased risk of ACVSD. RBM12 is an RNA-binding protein that has been reported to bind to the 5’ region of mRNA and regulate transcript splicing, mRNA localization, and translation.^35,49,50^ Moreover, unbiased screens have identified RBM12 as a repressor of GPCR (G-protein–coupled receptor)-cAMP–dependent signaling.^51^ Loss-of-function variants in RBM12 have not previously been linked to vascular risk. A previous study has reported PTVs in RBM12 to segregate with psychosis in an Icelandic and Finnish cohort but did not comment on ASCVD disease in this pedigree.^52^ The molecular function of SGTB is poorly characterized, but its homologue SGTA has a well-described role as a molecular chaperone.^33^ Our work provides impetus to elucidate the role of these proteins in vascular cells and to study their impact on atherosclerosis in model systems to provide orthogonal validation of these hypothesis-generating findings.

Our rationale for aggregating ASCVD across multiple vascular beds as a phenotype was 2-fold: (1) similar pathophysiological processes contribute to its development, and thus, power to detect genes driving common processes across vascular beds may be increased and (2) current paradigms for assessing efficacy of prevention and treatment of vascular disease typically assess capacity of interventions to reduce vascular risk across at least 2 vascular beds (typically coronary artery disease and cerebrovascular disease as part of major adverse cardiovascular events end point), so genes that increase risk across multiple vascular beds may be more attractive therapeutic targets. This approach was successful in the identification of 3 genes that seem to increase the risk of vascular disease independent of established vascular risk factors and 2 traits where the amalgamation of phenotypes resulted in exome-wide significant associations, which would not have been discovered if a single trait had been explored. However, it should be noted that there was evidence of heterogeneity of effects across vascular beds at a gene-burden test level and, as highlighted, at the variant level for HTRA1. Future studies could consider adopting analytical approaches that can account for this observed heterogeneity to maximize statistical power.

In summary, we have used a population genetics approach to identify novel genetic risk factors for ASCVD. We have provided evidence of heterogeneous effects of HTRA1 variants across vascular beds with concordant functional heterogeneity, suggesting a distinct mechanistic basis for HTRA1-related vascular disease in the coronary and cerebrovasculature.

## Article Information

### Sources of Funding

Dr Lockhart was funded by a Wellcome Trust Clinical PhD Fellowship (grant WT 225479/Z/22) and an Academic Clinical Lectureship from Queen’s University Belfast and the Department of Health, Northern Ireland. This work was funded by the Medical Research Council (Unit Program; grant MC_UU_00006/2). Dr Jackrel was supported by the National Institutes of Health grant R35GM153303.

### Disclosures

Dr O’Rahilly has undertaken remunerated consultancy work for Pfizer, Marea Therapeutics, Third Rock Ventures, AstraZeneca, NorthSea Therapeutics, and Courage Therapeutics. Dr Lockhart participates in paid consultancy for Eolas Medical. Drs Gardner and Perry are employees of Insmed Innovation UK and hold stock/stock options in Insmed. Dr Perry performs paid consultancy for WW International and receives research funding from GSK. The other authors report no conflicts.

### Supplemental Material

Supplemental Methods

Tables S1–S14

Figures S1 and S2

References 53–59

## Supplementary Material
