# Supplementary material for: Rare Variants in HTRA1, SGTB, and RBM12 Confer Risk of Atherosclerotic Cardiovascular Disease Independent of Traditional Cardiovascular Risk Factors
Source: Circ Genom Precis Med. 2025 Nov 5;18(6):e005233. doi: 10.1161/CIRCGEN.125.005233 (PMC7618418; doi:10.1161/CIRCGEN.125.005233)
Supplement: Supplementary file 1 [file hcg-18-e005233-s001.pdf]

Supplemental Material

Methods

Phenotype definitions

Atherosclerotic cardiovascular disease

We adapted a previously used definition of ASCVD[54] to assign case/control status to UK Biobank participants for clinically manifest ASCVD using a combination of medical record information, self-report information and death certificates. Briefly, case/control status for atherosclerotic cerebrovascular, coronary artery or peripheral vascular disease was defined using the self-report ICD9, ICD10 and OPSC4 terms listed in Supplementary Table S1. An individual with a qualifying term for any of these conditions was defined as a case, participants without any qualifying terms were defined as controls. Individual vascular bed phenotypes were defined by partitioning this phenotype into codes related to the coronary, cerebrovascular and peripheral circulation as defined in Table S1. Adaptations to the published definition[54] include the classification of individuals with angina (stable or unstable) as coronary artery disease cases. Also, participants undergoing surgical procedures or angioplasty typical used in the management of carotid atherosclerosis (e.g. carotid endarterectomy) as cerebrovascular disease cases. Unlike the index publication[54] which used only cerebrovascular and coronary artery disease to define ASCVD in their primary analysis, we considered any qualifying code related to the peripheral, coronary or cerebrovascular as indicative of ASCVD. Acute myocardial infarction was defined as the presence of an ICD10 code with the prefix I21 or an ICD9 code with the prefix 410 present in medical records or death certificate records linked to UKBB participants. Ischaemic stroke was similarly defined using the ICD10 prefix I63 and I64.

Cardiovascular risk factors

Phenotype definitions and processing details for a list of pre-specified cardiovascular risk factors are listed in Table S6.

Genetic association studies

Genetic association studies were conducted using available exome data from up to 434,438 UK Biobank participants of broadly European ancestry. Variant quality control, filtering, annotation and genetic association studies were conducted using a previously described bioinformatic pipeline - https://github.com/mrcepid-rap[14-16].

Variant filtering and quality control

Exome sequence data was subject to quality control and variant filtering as described previously [55]. Further to this, multi-allelic variants in processed VCF files were split and left-corrected into separate alleles before filtering as previously described[16]. Briefly, single nucleotide variants were excluded if depth <7 and genotype quality <20 and InDels were excluded if depth<10 and or genotype quality <20. Heterozygous variants with alternate allele reads significantly different that the expect 50% (Binomial P-value

<0.001) were excluded as were variants where missingness after the above steps exceeded 50%.

Variant annotation and gene-burden mask definitions

Variants were annotated with Ensembl variant effect predictor[56], including the LOFTEE plugin[21] and REVEL[22]. For each gene, rare (MAF<0.001), high confidence protein truncating variants as defined by LOFTEE and rare (MAF<0.001) missense variants with either REVEL>0.5 or REVEL>0.7 were collapsed into masks for the purposes of burden testing. The use of variant annotation methods including both protein truncating and missense variants is intended to maximise chance of discovery. For example, missense masks may be useful where loss of function PTVs do not provide adequate power or where more complex functional effects drive a phenotype (e.g. gain of function variants, dominant negative effects). Only masks with sufficient carriers to be informative (MAC>30) were retained. This process resulted in 37,686 individual gene x mask combinations and a stringent, Bonferroni-corrected multiple testing threshold of $P<1.33 \times 10^{-6}$. A mask of rare (MAF<0.0001) synonymous variants was derived and used as a negative control.

Association testing

Association testing for ASCVD, coronary artery disease, peripheral vascular disease and cerebrovascular disease was conducted using a Linear Mixed Model implemented using BOLT-LMM to account for cryptic relatedness and population stratification as previously described[16,57]. Age, sex, age^2, sequencing batch and the first 10 genetic principal components were included in the model as co-variates. Generalised linear models were used to derive odds ratios, as effect estimates generated from linear mixed models are reported on the quantitative scale, rather than the log(OR) scale which is more intuitive to interpret. Odds ratio estimation by generalised linear models was conducted using the python package ‘statsmodels’ with family set to binomial. Relatedness was not accounted for. Phenotypic testing to assess the effects of exome wide significant gene-burden masks on cardiovascular risk factors was implement in STAAR[58] using the ‘extract’ function in the ‘run association testing’ applet, with adjustment for the co-variates stated above. Sensitivity and follow-up analyses related to the effects of R227W variants in HTRA1 were performed using either gene burden testing implemented in STAAR using the ‘extract’ function in the ‘run association testing’ applet or using generalised linear models implemented in R adjusted for the aforementioned co-variates. As an additional assessment of the robustness of our results we conducted gene burden testing using SAIGE-GENE+, this was conducted using the exact same samples and covariates used in discovery.

Bespoke pathogenic variant NOTCH3 gene burden testing

To assess the effect of pathogenic NOTCH3 variants on cerebrovascular disease and coronary artery disease we generated a bespoke mask for gene burden testing using the ‘collapse-variants’ applet in the MRC-Epidemiology RAP association testing pipeline to collapse a previously described list of curated pathogenic NOTCH3 variants[29]. Association testing for a relationship with cerebrovascular and coronary artery disease was conducted using STAAR as described above.

HTRA1 activity score analyses

To assess for a relationship between experimentally characterised HTRA1 protease activity and cerebrovascular disease, coronary artery disease and ischaemic stroke we used proteolytic activity as reported previously[40], as the predictor variable in a logistic regression analysis adjusted for age, age^2, sex, genetic PC1-10 and exome sequencing batch and sensitivity to the inclusion/exclusion of R227W was assessed. Of the 76 variants with reported protease activity, 73 were present in our filtered dataset and included in our analysis.

Assessment of phenotypic heterogeneity of predicted damaging HTRA1 variants

To formally demonstrate the heterogenous effects of R227W and other predicted damaging missense variants in HTRA1 across cerebrovascular and coronary artery disease we took two approaches. Firstly we conducted logistic regression analyses for the R227W variant and rare, predicted damaging (MAF<0.001, REVEL>0.5) missense variants in HTRA1 excluding R227W and then tested for heterogeneity between effect estimates using Cochrane's Q-test for heterogeneity in the *metafor* package. Secondly, we compared the effects of carriage of the R227W variant on cerebrovascular and coronary artery disease risk compared to other carriers of rare, predicted damaging (MAF<0.001, REVEL>0.5) missense variants in HTRA1.

Vascular expression of ASCVD risk genes

To determine if vascular risk genes were expressed and differentially regulated in the vascular wall of participants with or without atherosclerosis we queried the STARNet database[38]. Exome wide significant genes were queried and relevant summary statistics extracted.

Functional studies

Protein purification

All proteins were expressed and purified from *E. coli* BL21-CodonPlus(DE3)-RIL cells (Agilent) and purified under native conditions, as previously described[41]. Briefly, plasmid containing the HTRA1 (residues 156-480) with a C-terminal 6-His tag in the pET21a plasmid was obtained from the Saghatelian lab [59]. All missense mutants were generated by site-directed mutagenesis, with sequences confirmed by Sanger sequencing. To generate recombinant protein, *E. coli* cells were induced at OD_{600}=0.6 with 0.4mM IPTG for 18h at 16ºC. Cell pellets were resuspended in HTRA wash buffer (50mM Tris, pH 8.0, 1M NaCl, and 30mM imidazole) supplemented with lysozyme (20mg per L of initial culture) and protease inhibitors (cOmplete, EDTA free, Roche). Cells were lysed by sonication and the lysate was cleared by centrifugation. The supernatant was then incubated with Fast flow nickel sepharose (GE Healthcare) for 2h at 4ºC, and all subsequent steps occurred at 4ºC. The resin was then transferred to a column, washed with HTRA wash buffer, and eluted with HTRA elution buffer (50mM Tris, pH 8.0, 100mM NaCl, and 500mM imidazole). The protein was then buffer exchanged into HTRA storage buffer (50mM Tris, pH 8.0, 100mM NaCl, 10% glycerol) and concentrated to approximately 5-10mg/mL. The protein was then flash frozen in liquid nitrogen before

storage at -80ºC. Protein was stored in the freezer for no longer than three months to minimize auto-proteolysis.

Plasmids for expression of α-synuclein were from Peter Lansbury[60]. α-Syn was expressed in *E. coli* BL21-DE3-RIL cells (Invitrogen), where expression was induced at OD_{600}=0.6 with 1mM IPTG for 2h at 37ºC. Cell pellets were resuspended in osmotic shock buffer (30mM Tris, pH 7.2, 2mM EDTA, 40% sucrose). Cells were lysed by incubating in osmotic shock buffer for 10min at room temperature, centrifuged, and resuspended in 0.84mM $MgCl_2$. Lysate was then cleared by centrifugation. Nucleic acids were removed via streptomycin sulfate precipitation. The supernatant was then boiled for 10min, after which most proteins precipitate while α-syn remains soluble following boiling. Protein was then loaded onto a bed of DEAE sepharose for anion-exchange. The column was washed with wash buffer (20mM Tris, pH 8, 1mM EDTA) and eluted with elution buffer (20mM Tris, pH 8.0, 300mM NaCl, 1mM EDTA). The eluate was then dialyzed into α-syn fibrillization buffer (20mM Tris, pH 8.0, 100mM NaCl), flash frozen, and stored at -80ºC until use.

Proteolysis assay:
FITC-casein (10μM) was treated with HTRA1 and its variants (2μM) for 2.5h at 25°C in buffer (20mM Tris, pH 8.0, 100mM NaCl). Degradation of FITC-casein was monitored at 535nm after excitation at 485nm using a Tecan Spark plate reader. Casein (40μM) was incubated with buffer (20mM Tris, pH 8.0, 100mM NaCl), HtrA1 and its variants (2.5μM) for 24h at 37°C. Samples were collected at 0h and 24h and then processed by SDS-PAGE. α-Synuclein (25μM) was incubated with buffer (20mM Tris, pH 8.0, 100mM NaCl), HtrA1 and its variants (5μM) for 24h at 37°C. Samples were collected at 0h and 24h and then processed by SDS-PAGE.

α-Synuclein inhibition assay:
For α-synuclein inhibition assays, α-synuclein monomer (25μM) was incubated in buffer (20mM Tris, pH 8.0, 100mM NaCl), with and without the indicated HTRA variants (5μM) for 72h at 37°C with agitation of 1500 rpm in a thermomixer. Samples were mixed with ThioflavinT (ThT) dye to quantify α-synuclein aggregation. Fluorescence at 482nm was measured after excitation at 450nm using a Tecan Spark plate reader. For sedimentation assays, samples were collected at 72h and centrifuged at 15000 rpm for 30 min at room temperature to separate soluble and pellet fractions. Samples were mixed with sample buffer (60mM Tris, pH 6.8, 5% glycerol, 2% SDS, 4% β-mercaptoetanol) at a 3:1 ratio. The samples were boiled and separated on SDSPAGE and stained with Coomassie Brilliant Blue.

All in vitro data presented is the mean of or is representative of results from at least 3 independent experiments.

Supplemental Tables: (Separate Excel)

Table S1: Phenotype definitions

Table S2. Test statistics from an exome wide association study of ASCVD in UKBB, only exome-wide significant associations are shown

Table S3: Leave one out analyses

Table S4: Sensitivity to variation in phenotype definition

Table S5. Test statistics from an exome wide association study of atherosclerotic cardiovascular disease across vascular bed

Table S6: Phenotype definitions for assessed cardiovascular risk factors

Table S7. Effect of ASCVD risk genes on continuous cardiovascular risk factors

Table S8. Effect of ASCVD risk genes on binary cardiovascular risk factors

Table S9. Effects of novel ASCVD risk genes on ASCVD after adjustment for traditional cardiovascular risk factors (LDL-C, BMI, SBP, T2DM, Smoking)

Table S10. Effects of pathogenic variants in NOTCH3 on coronary and cerebrovascular disease

Table S11: Differential effects of R227W on cerebrovacular and coronary artery disease

Table S12: Effects of exclusion of R227W on the relationship between HTRA1 protease activity and vascular disease

Table S13. List of diagnostic codes used

Table S14. Comparison of burden test results using different tools

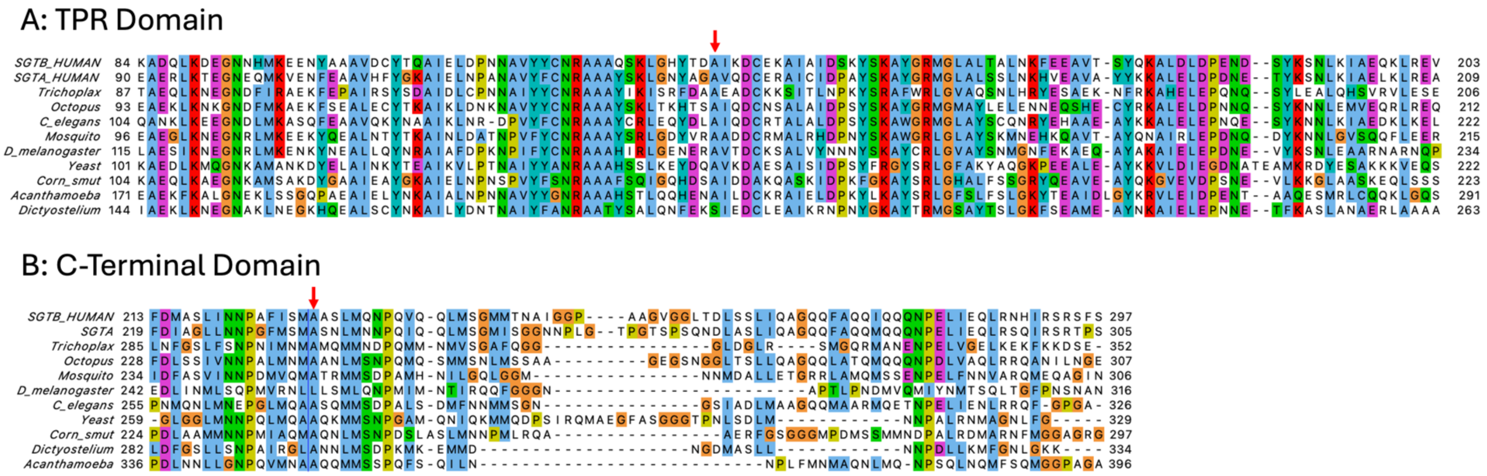

Figure S1 Missense mutations in SGTB associated with ASCVD risk are highly conserved. Representative alignments of portions of the TPR (A) and C-Terminal domain (B)of SGTB. Given the high levels of conservation of these regions throughout evolution, remote paralogues have been chosen to show diversity. The red arrows in A and B represent the p.SGTB A138V and A228V mutations in the TPR and C-terminal domain, respectively.

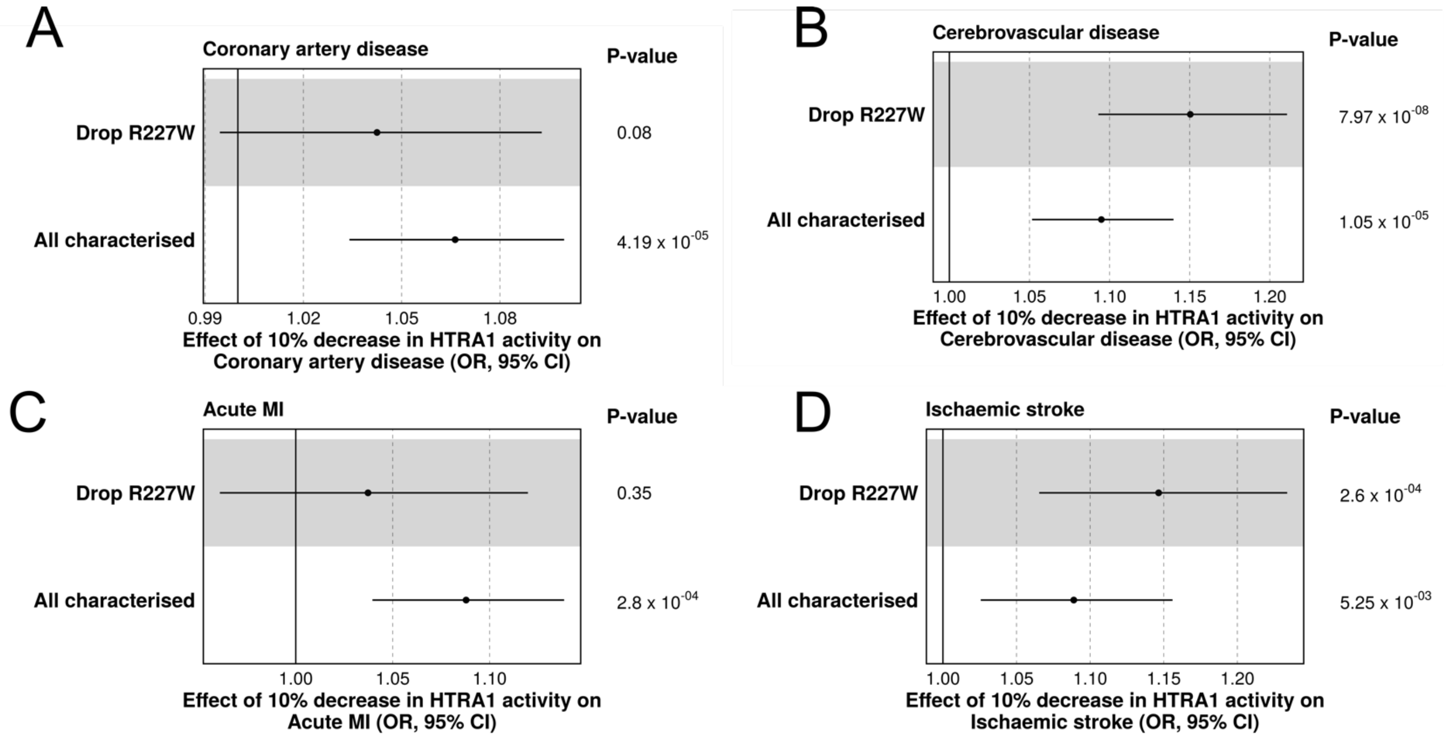

Figure S2 Sensitivity of an experimentally derived HTRA1 protease score on inclusion/exclusion of R227W*:* The effects of R227W inclusion/exclusion on association of an experimentally derived HTRA1 activity score and vascular disease risk on risk of coronary artery disease (A) cerebrovascular disease (B), acute myocardial infarction (C) and ischaemic stroke (D). OR=Odds ratio CI= Confidence Interval. Test statistics plotted and P-values were derived from a generalised linear model.
